# Time to Total Hip Arthroplasty Among Patients in the US Military Health System

**DOI:** 10.1001/jamanetworkopen.2025.39971

**Published:** 2025-10-28

**Authors:** Brandon L. Hillery, Ashton H. Goldman, Alexander G. Velosky, Maxwell Y. Amoako, Jeffrey C. Leggit, Krista B. Highland

**Affiliations:** 1 School of Medicine, Uniformed Services University, Bethesda, Maryland; 2 Department of Orthopedic Surgery, Naval Medical Center Portsmouth, Portsmouth, Virginia; 3 Department of Surgery, Uniformed Services University, Bethesda, Maryland; 4 Defense and Veterans Center for Integrative Pain Management, Department of Anesthesiology, Uniformed Services University, Bethesda, Maryland; 5 Henry M. Jackson Foundation for the Advancement of Military Medicine Inc, Bethesda, Maryland; 6 Enterprise Intelligence and Data Solutions Program Office, Program Executive Office, Defense Healthcare Management Systems, San Antonio, Texas; 7 Department of Family Medicine, Uniformed Services University, Bethesda, Maryland; 8 Department of Anesthesiology, Uniformed Services University, Bethesda, Maryland

## Abstract

**Importance:**

Total hip arthroplasty (THA) delays can be deleterious. Robust evaluation of time to THA enables data-driven improvement efforts across the US Military Health System.

**Objective:**

To evaluate time to THA after hip osteoarthritis diagnosis by patient-, care-, and structural-level factors.

**Design, Setting, and Participants:**

This retrospective cohort study evaluated medical records from US military and civilian health care facilities from March 1, 2015, to June 21, 2024. Participants were adult TRICARE enrollees diagnosed with hip osteoarthritis between March 1, 2018, and March 30, 2023, without diagnosis in the past 3 years, who received at least 1 additional visit 1 week to 3 years after the index date. Data were analyzed from July 2024 to August 2025.

**Main Outcomes and Measures:**

Time to THA within 3 years of index diagnosis.

**Results:**

Of 37 239 patients diagnosed with hip osteoarthritis (median [IQR] age, 59 [50-64] years; 21 553 [58%] male; 320 American Indian and Alaska Native [1%]; 1603 Asian and Pacific Islander [4%]; 8123 Black [22%]; 2041 Hispanic [5%]; 23 327 White [63%]; 1825 another race and ethnicity [5%]), 10 502 (28%) received a THA within 3 years. A piecewise exponential additive model indicated incidence rate ratios (IRRs) for time to THA were lower for Asian and Pacific Islander (IRR, 0.76; 95% CI, 0.66-0.88), Black (IRR, 0.79; 95% CI, 0.74-0.85), and Hispanic (IRR, 0.84, 0.73-0.96) patients compared with White patients; patients with psychiatric (IRR, 0.79; 95% CI, 0.76-0.83), pain-related (IRR, 0.69; 95% CI, 0.66-0.72), or obesity (IRR, 0.92; 95% CI, 0.88-0.96) diagnoses before the index diagnosis; patients with elevated comorbidity index scores (IRR, 0.85; 95% CI, 0.74-0.97); those diagnosed in the purchased care system (IRR, 0.38; 95% CI, 0.36-0.40) and in later years (IRR, 0.97; 95% CI, 0.95-0.99); and patients with 1 (IRR, 0.83; 95% CI, 0.77-0.90) or more (IRR, 0.75; 95% CI, 0.70-0.81) injections or 2 or more imaging visit days (IRR, 0.77; 95% CI, 0.69-0.86) before the index diagnosis. Retired service members (IRR, 1.39; 95% CI, 1.24-1.56) and family members (IRR, 1.48; 95% CI, 1.30-1.69) had higher incidence of THA compared with active-duty service members. Male patients had higher incidence compared with female patients (IRR, 1.20; 95% CI, 1.11-1.31). Patients with increasing orthopedic surgeon visit days (IRR, 1.67; 95% CI, 1.64-1.69), opioid prescriptions (IRR, 1.02; 95% CI, 1.01-1.03), and imaging visit days (IRR, 1.26; 95% CI, 1.24-1.28) after the index diagnosis were also more likely to undergo THA. Variation across geographic areas was mixed.

**Conclusions and Relevance:**

In this cohort study of TRICARE enrollees, variation in THA receipt was identified across multilevel factors. These finding suggest that policy and programming decisions could benefit from including TRICARE-participating orthopedic surgeon locations, structural factors, and reimbursement models to optimize THA access.

## Key Points

**Question:**

How does time to total hip arthroplasty (THA) after hip osteoarthritis diagnosis vary across patient-, care-, and structural-level factors in the US Military Health System?

**Findings:**

In this cohort study including 37 239 patients, time-to-THA varied across patient (eg, age, race and ethnicity, sex, beneficiary category), care (eg, imaging, orthopedic surgeon visits, injections, therapeutic visits), and structural (eg, health care system, year) factors.

**Meaning:**

These results highlight variation in THA timing across military and civilian health care systems; future data-driven policy and programming may optimize timely care.

## Teaser

This cohort study assesses variations in time to total hip arthroplasty after hip osteoarthritis diagnosis among patients in the US Military Health System.

## Introduction

Delays in total joint arthroplasty are associated with decreased quality of life and may confer worse postarthroplasty outcomes.^1^ While the projected demand for total hip arthroplasty (THA) is estimated to increase over the next decades,^2,3^ access is tempered by variation in orthopedic surgeon location and training programs.^4,5^ To date, research has identified several patient- and structural-level factors associated with THA receipt across multi-institutional studies, national samples, and Military Health System (MHS), Veterans Health Administration, and Medicare and Medicaid beneficiaries.^6,7,8,9,10,11,12,13^ However, there is a lack of literature that accounts for nonsurgical guideline-congruent and guideline-incongruent care after osteoarthritis diagnosis (eg, opioid prescriptions, imaging, therapeutic interventions)^14,15,16,17,18,19^ and uses time-to-event models to account for longitudinal nuances in hip osteoarthritis care.

Given delayed and inadequate osteoarthritis treatment can negatively impact quality of life and incur financial burden,^20^ evaluating THA timing within the context of osteoarthritis-related care enables targeted intervention across large health care systems. In the MHS, patients receiving THA tend to be younger and male relative to national databases.^21^ Moreover, unlike private health care settings, surgeons in military treatment facilities do not contend with payers or receive increased reimbursement based on productivity. Patients enrolled in TRICARE insurance (eg, active duty and retired service members and their eligible family members) do not incur large out-of-pocket costs when receiving a THA within military treatment facilities (eg, direct care system) but may pay more in the civilian network (eg, purchased care system). Therefore, evaluating THA timing in MHS beneficiaries enables evaluation of the context of direct and purchased care systems.

The objective of this study was to evaluate time to THA after hip osteoarthritis diagnosis in patients enrolled in insurance plans in which some or all health care is provided at military treatment facilities, when such services are available at the local facility (eg, TRICARE Prime, TRICARE Plus, direct care only). These plans are like health maintenance organization plans in which patients must receive specialty care referrals (eg, orthopedic surgery, imaging) from their primary care managers. Other plans that are outside of the scope of this study, such as TRICARE Select, are akin to preferred provider organizations and incur higher out-of-pocket costs. It was hypothesized that variation in time to THA would be associated with patient-, care-, and structural-level factors, which would be maintained in sensitivity models evaluating patients receiving care in only the direct vs purchased care systems, separately. Given prior research, it was specifically hypothesized that THA receipt would be less likely in American Indian and Alaska Native, Asian and Pacific Islander, Black, and Hispanic patients and patients with another race and ethnicity compared with White patients; female patients compared with male patients; older patients compared with younger patients; and patients with fewer imaging, orthopedic surgeon, and therapeutic visits compared with those with more visits.

## Methods

### Study Design, Data Sources, and Record Selection

This retrospective cohort study was determined to be exempt from review and informed consent by the Uniformed Services University institutional review board because this study leveraged secondary research for which consent is not required, per the Revised Common Rule, 45 CFR 46.104(d)(4). This study followed the Strengthening the Reporting of Observational Studies in Epidemiology (STROBE) reporting guideline. Medical record data (eg, encounter records, prescriptions) were extracted from the MHS Information Platform. Records were included of adult patients ages 18 to 89 years, who received an *International Statistical Classification of Diseases and Related Health Problems, Tenth Revision *(*ICD-10*) M16 diagnosis for hip osteoarthritis (primary, posttraumatic, and unspecified osteoarthritis) between March 1, 2018, and June 30, 2023 (ie, index diagnosis), without prior documented diagnosis from March 1, 2015, to March 29, 2018, to ensure this diagnosis was the first within a 3-year period. There was no requirement that the hip osteoarthritis diagnosis be the primary diagnosis for the encounter.

Patients were excluded if they had an M16 diagnosis within 3 years prior to the index hip osteoarthritis diagnosis (including *International Classification of Diseases, Ninth Revision *[*ICD-9*] and *ICD-10* diagnosis codes) or THA; did not have race and ethnicity documented in their medical records; were not an active-duty service member, retired service member (eg, served ≥20 years before separation), or family member; were not enrolled in TRICARE Prime, TRICARE Plus, or direct care only plans at index; had a diagnosis corresponding to cancer, infection, or hip fracture between index and censor dates; or lacked documented hip osteoarthritis diagnoses after the week of index diagnosis, as time-to-event analyses required censoring or the terminal event (THA receipt) to occur after the index (ie, week 0) and data were aggregated on the week-level. Therefore, all patients had at least 1 follow-up visit in which hip osteoarthritis was documented at least 1 week and up to 3 years after index. After identifying the index diagnosis, direct and purchased care records up to 3 years before and 3 years after the index diagnosis were extracted up until June 21, 2024. As such, some patients did not have 3 years of postindex records. The Figure provides a flowchart of included and excluded records.

**Figure.**
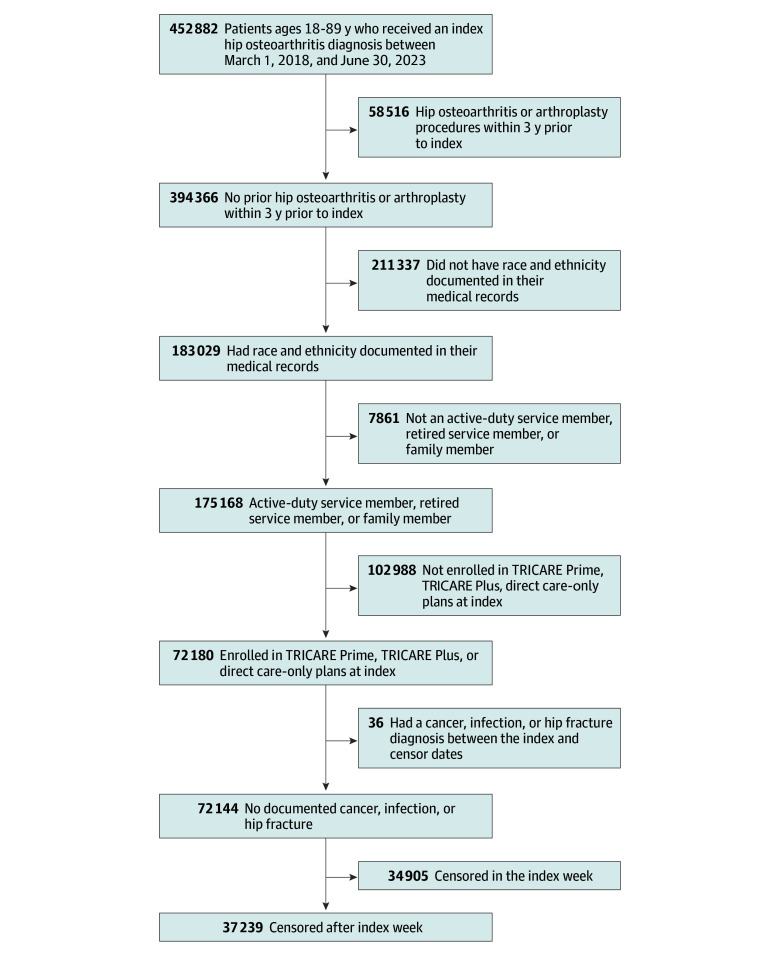
Flowchart of Patient Selection and Inclusion

### Primary Outcome

The primary outcome was time-to-THA receipt within 3 years after the index diagnosis. Receipt of THA was indicated by Current Procedural Terminology code 27130 or *ICD-10* Procedure Coding System codes 0SR9*, 0SRA*, 0SRB*, 0SRE*, 0SRR*, or 0SRS*.

### Model Covariates

Covariates included patient-, care-, and structural-level factors. Patient-level information included self-reported race and ethnicity recorded in the administrative records (American Indian and Alaska Native, Asian and Pacific Islander, Black, Hispanic, White, and another race and ethnicity [eg, >1 race and ethnicity]), sex (male or female), age, and beneficiary group (active-duty service member, retired service member, family member). Age was retained as a continuous variable in the models and categorized for descriptive purposes into 3 age groups: younger than 55 years, 55 to 69 years, and 70 years or older. Patient-level diagnosis covariates included those documented within 1 year before the index diagnosis. Diagnosis covariate flags (yes or no) included psychiatric diagnoses, pain-related diagnoses per prior literature,^22^ and obesity diagnoses per prior literature.^23^ An age-adjusted Charlson Comorbidity Index score^24^ was categorized as 0, 1, or 2 or greater. Tobacco use (yes or no) was defined as nicotine dependence diagnosis or self-reported and clinician-documented tobacco use. Care-related covariates included hip injections and hip imaging visits before index diagnosis using procedure codes described in prior studies^25^ (eAppendix in Supplement 1). Due to data skewness, 3-year preindex cumulative hip injections and hip imaging receipt were categorized as 0, 1, or 2 or more visits, each. Additional care-related covariates were time-dependent and cumulative, such that values could increase across time and had a maximum value of the 99th percentile due to data skewness. These covariates (99th percentile) included postindex cumulative opioid prescriptions (9 prescriptions) and days in which patients received hip imaging (7 days), orthopedic surgeon visits (6 days), and therapeutic visits (eg, physical therapy, biofeedback injections, other interventional pain therapies; 24 days). Hip injections and opioid prescriptions may have been for other pain-related conditions but could have contributed to hip osteoarthritis management. We used treatment days rather than encounters because some encounters included multiple procedure codes (eg, manual therapy, education, exercise therapy) and clinicians (eg, physical therapist, occupational therapist). At the index week, all postindex cumulative variables were set at 0, then increased by 1 with each care-related event. Lastly, structural-level information included the index care system (direct, purchased), year of index hip osteoarthritis diagnosis, and geographic area of index diagnosis per US Census regions and divisions.^26^ Divisions are a more specific geographic area and were used unless sample size warranted use of the region. Therefore, geographic area categories were Pacific (division), Mountain (division), Midwest (region), Northeast (region), West South Central (division), East South Central (division), South Atlantic (division), and in US Territories, in another country, or in an unknown location (neither a division nor region).

### Statistical Analysis

Records were right-censored on the last recorded visit date if no THA occurred and no visits occurred after the 3-year postindex period or at 3 years after index if no THA occurred and visit records continued after 3 years. Nonparametric bivariate analyses (eg, Kruskal-Wallis tests, nonparametric χ^2^ tests) with pairwise tests and Benjamini and Hochberg–adjusted *P* values compared patients who (1) were excluded due not having race and ethnicity recorded in administrative records, as well as due to censoring in the index week (eg, had no other hip osteoarthritis visits after 1-week after the index diagnosis) vs included in the analysis (eTable 1 in Supplement 1), (2) those who did not and did receive a THA within 3 years after the index diagnosis, and (3) those who received hip osteoarthritis care in the purchased care system only, direct system only, or both systems (eTable 2 in Supplement 1). Bivariate tables were constructed using the compareGroups^27^ package in R software versions 4.4.0 to 4.5.1 (R Project for Statistical Computing). We used the survminer package^28^ in R to create unadjusted survival curves and a censoring table across races and ethnicities (eFigure 1 in Supplement 1).

The primary model was constructed in 2 steps. First, a Cox proportional hazards model (survival model) examined the assumption of proportionality for each covariate using the survival package^29^ in R. The proportionality assumption assumes the hazard ratio is constant across the observation period and all covariate levels. Covariates not meeting the proportionality assumption were modeled as fixed and time-varying covariates (eg, time × covariate interaction) in subsequent models.

The primary model was a piecewise exponential additive model, a generalized additive model (GAM) with a Poisson distribution, estimated using the pammtools^30^ and mgcv^31^ R packages. With GAM, continuous covariates with adequate ranges (eg, age) can be modeled as smooth covariates to model nonlinear associations between covariates and the outcome. Time-varying covariates are modeled as smooth covariates to allow for effects that can change across time (eg, nonproportional hazards). When time-varying covariates are not significant, the corresponding fixed covariates are interpreted alone. If corresponding fixed and time-varying covariates are significant, the fixed covariate indicates the geometric mean baseline hazard and the time-varying covariate is interpreted using visualizations to determine how the hazards change across time.^32^

Three sensitivity models were evaluated. Two GAMs replicated the primary model separately for patients who received hip osteoarthritis care solely in direct vs purchased care systems. A third GAM extended the direct care–only GAM by including a random effect for military treatment facility of index diagnosis. Due to sample size requirements with multilevel models, sites with at least 25 index diagnoses were included.

GAM results are rendered as incidence rate ratios (IRRs) with 95% CIs, which indicate the incidence of THA in a comparator group relative to the incidence in the reference group. For example, if the incidence rate was 10% in a comparison group and 20% in a reference group, the IRR would be 0.5 (10% / 20%). Results of the GAM were tabulated using the sjPlot R package.^33^ Estimated survival probabilities for fixed, smooth, and time-varying covariates were calculated using the pammtools R package^30^ based on the a priori reference categories of nonfocal variables as follows: White race and ethnicity, age 60 years, male, active-duty service member, with an age-adjusted CCI score of 1, no psychiatric or obesity diagnosis prior to the index diagnosis, with a pain-related diagnosis, index direct care system, index year 2020, index diagnosis in the South Atlantic geographic area, 0 hip injections or imaging visits prior to the index diagnosis, 1 imaging visit after diagnosis, 0 opioid prescriptions after diagnosis, and 2 therapeutic visits after diagnosis. These probabilities facilitate inferences regarding relative differences. Changes to a priori categories shift probability estimates. The cumulative hazard differences for significant time-varying smoothed covariates were calculated using the pammtools R package.^30^ Time-varying survival probabilities and cumulative hazard differences were visualized using the ggplot2 R package.^34^ *P* values were 2-sided, and statistical significance was set at *P* ≤ .05. Data were analyzed from July 2024 to August 2025.

## Results

### Sample Description

Overall, 37 239 patients (median [IQR] age 59 [50-64] years; 21 553 [58%] male) met all inclusion and exclusion criteria (Figure and Table 1). The cohort included 320 American Indian and Alaska Native patients (1%), 1603 Asian and Pacific Islander patients (4%), 8123 Black patients (22%), 2041 Hispanic patients (5%), 23 327 White patients (63%), and 1825 patients with another race and ethnicity (5%). Most patients had an index diagnosis for primary hip osteoarthritis (33 659 patients [90%]), followed by unspecified (2383 patients [6%]), hip dysplasia–related (578 patients [2%]), posttraumatic (389 patients [1%]), other (224 patients [1%]), and secondary (92 patients [<1%]) osteoarthritis. More than half of patients (20 821 patients [56%]) received all hip osteoarthritis care (eg, index and follow-up care) in the purchased care system only; 6896 patients (19%) received only direct system care for hip osteoarthritis; the remaining 9522 patients (26%) received hip osteoarthritis care across both systems. The median (IQR) proportion of hip osteoarthritis care provided in the direct care system was 0% (0%-64%) and the mean (SD) was 29% (40%). Of patients who received a THA, 9048 patients (86%) had a THA on the side matching their index diagnosis; 1113 patients (11%) had a THA on the side matching a postindex diagnosis; and 341 patients (3%) had an unspecified THA and/or hip osteoarthritis side or were unmatched in laterality.

**Table 1.** Sample Descriptive Information and Bivariate Analyses Between Patients Who Did Not vs Did Receive a THA Within 3 Years of Index Hip Osteoarthritis Diagnosis

Characteristic	Patients, No. (%)			*P* value
	Overall (N = 37 239)	Received THA		
		No (n = 26 737)	Yes (n = 10 502)	
Age, median (IQR), y	59 (50-64)	58 (48-64)	60 (54-65)	<.001
Race and ethnicity				
American Indian and Alaska Native	320 (1)	248 (1)	72 (1)	<.001
Asian and Pacific Islander	1603 (4)	1276 (5)	327 (3)	
Black	8123 (22)	6296 (24)	1827 (17)	
Hispanic	2041 (5)	1603 (6)	438 (4)	
White	23 327 (63)	15 921 (60)	7406 (71)	
Another race and ethnicity	1825 (5)	1393 (5)	432 (4)	
Beneficiary group				
Active-duty service member	5730 (15)	4843 (18)	887 (8)	<.001
Family member	13 229 (36)	9703 (36)	3526 (34)	
Retired service member	18 280 (49)	12191 (46)	6089 (58)	
Sex				
Female	15 686 (42)	11 717 (44)	3969 (38)	<.001
Male	21 553 (58)	15 020 (56)	6533 (62)	
Age-adjusted CCI category				
0	7227 (19)	5985 (22)	1242 (12)	<.001
1	8151 (22)	5595 (21)	2556 (24)	
≥2	21 861 (59)	15 157 (57)	6704 (64)	
Diagnosis 1 y before index date				
Psychiatric diagnosis	9957 (27)	7776 (29)	2181 (21)	<.001
Pain-related diagnosis	31 285 (84)	23 076 (86)	8209 (78)	<.001
Obesity	8575 (23)	6189 (23)	2386 (23)	.39
Nicotine dependence or documented tobacco use	4277 (11)	3189 (12)	1088 (10)	<.001
Index diagnosis system				
Direct	13 120 (35)	9000 (34)	4120 (39)	<.001
Purchased	24 119 (65)	17 737 (66)	6382 (61)	
Index year, median (IQR)	2021 (2019-2022)	2021 (2019-2022)	2021 (2019-2022)	<.001
Care received 3 y before index date				
Hip injections				
0	31 683 (85)	22 609 (85)	9074 (86)	<.001
1	2599 (7)	1925 (7)	674 (6)	
≥2	2957 (8)	2203 (8)	754 (7)	
Hip imaging				
0	28 085 (75)	20 170 (75)	7915 (75)	.01
1	6092 (16)	4310 (16)	1782 (17)	
≥2	3062 (8)	2257 (8)	805 (8)	
Geographic region				
East South Central	2601 (7)	1885 (7)	716 (7)	<.001
Midwest	2873 (8)	1998 (7)	875 (8)	
Mountain	3489 (9)	2323 (9)	1166 (11)	
Northeast	1196 (3)	878 (3)	318 (3)	
Pacific	4830 (13)	3302 (12)	1528 (15)	
South Atlantic	15 059 (40)	10 779 (40)	4280 (41)	
US territory, another country, unknown	947 (3)	819 (3)	128 (1)	
West South Central	6244 (17)	4753 (18)	1491 (14)	

Abbreviations: CCI, Charlson Comorbidity Index; THA, total hip arthroplasty.

Across the 3-year postindex period and without accounting for censoring, 502 patients (28%) received a THA. Proportions were highest among White patients (7406 patients [32%]), followed by patients with another race and ethnicity (432 patients [24%]), and American Indian and Alaska Native (72 patients [23%]), Black (1827 patients [23%]), Hispanic (438 patients [22%]), and Asian and Pacific Islander (327 patients [20%]) patients. Of patients who received a THA, the unadjusted time to THA was lowest for Hispanic patients (median [IQR], 28 [14-59] weeks), followed by White patients (median [IQR], 29 [14-65] weeks), patients with another race and ethnicity (median [IQR], 31 [14-68]), Asian and Pacific Islander patients (median [IQR], 34 [15-69] weeks), Black patients (median [IQR], 36 [16-77] weeks), and American Indian and Alaska Native patients (median [IQR], 44 [15-81]). Proportions were higher and time to THA lower for male (6533 patients [30%]; median [IQR], 29 [14-65] weeks) relative to female (3969 patients [25%]; median [IQR], 32 [15-70] weeks) patients and for retired service members (6089 patients [33%]; median [IQR], 29 [13-62] weeks) and family members (3526 patients [27%]; median [IQR], 33 [15-71] weeks) compared with active-duty service members (887 patients [16%]; median [IQR], 38 [17-78] weeks). Patients younger than 55 years had the lowest proportions of unadjusted THA receipt (13 325 patients [21%]), followed by patients 70 years and older (6098 patients [28%]) and ages 55 to 69 years (17 816 patients [34%]). However, patients 70 years and older had the shortest time to THA (median [IQR], 27 [13-60] weeks) compared with patients ages 55 to 69 years (median [IQR], 30 [14-66] weeks) and younger than 55 years (median [IQR], 34 [15-74] weeks). Patients receiving a hip osteoarthritis diagnosis in the direct care system had both higher proportion of THA receipt (4120 patients [31%]) and longer time to THA (median [IQR], 34 [16-71] weeks) than in the purchased care system (6382 patients [27%]; median [IQR], 28 [13-64] weeks). Lastly, THA receipt was highest and time to THA shortest in the Mountain area (1166 patients [33%]; median [IQR], 27 [13-62] weeks), whereas THA occurred least often and with the longest duration for patients in US territories, in other countries, or in an unknown location (128 patients [14%]; median [IQR], 45 [23-88] weeks). Unadjusted survival curves and censoring frequencies by race and ethnicity, sex, beneficiary group, age group, system of diagnosis, and geographic area are in eFigure 1 in Supplement 1.

Bivariate analysis indicated patients included vs excluded varied across all tested variables (eTable 1 in Supplement 1). All potential covariates, aside from an obesity diagnosis, were significantly different between patients who did not and did receive a THA (Table 1). In the Cox proportional hazards models, several variables across both primary and sensitivity models were not proportional and therefore were included as time-varying covariates.

### Primary Model

Time to THA varied across several patient-, care-, and system-level covariates. Asian and Pacific Islander patients (IRR, 0.76; 95% CI, 0.66-0.88; *P* < .001), Black patients (IRR, 0.79; 95% CI, 0.74-0.85; *P* < .001), and Hispanic patients (IRR, 0.84; 95% CI, 0.73-0.96; *P* = .01) had lower incidence of THA compared with White patients across time (Table 2; eFigure 4 in Supplement 1). There was a significant time-varying effect for Black patients, such that cumulative hazard differences steeply decreased within the first year, with less intense decreases afterward. Adjusted probabilities indicated that an estimated 37% (95% CI, 32%-42%) of White patients received a THA by 3 years after the index diagnosis, followed by Hispanic patients (34%; 95% CI, 29%-40%), patients with another race and ethnicity (33%; 95% CI, 28%-39%), American Indian and Alaska Native patients (32%; 95% CI, 24%-42%), Asian and Pacific Islander patients (30%; 95% CI, 25%-36%), and Black patients (28%; 95% CI, 24%-33%). To examine how a priori levels of nonfocal covariates might affect estimates, probabilities were recalculated to change the assumption of direct care index diagnosis to purchased care. Adjusted probabilities maintained the same order, with White patients having the highest estimated probability of THA at 3 years (18%; 95% CI, 16%-21%), followed by Hispanic patients (17%; 95% CI, 14%-20%), patients with another race and ethnicity (16%; 95% CI, 13%-19%), American Indian and Alaska Native patients (15%; 95% CI, 11%-21%), Asian and Pacific Islander patients (15%; 95% CI, 12%-18%), and Black patients (13%; 95% CI, 11%-16%). Adjusted probabilities across races and ethnicities are depicted in eFigure 2 in Supplement 1.

**Table 2.** Primary Poisson Generalized Additive Model Estimating Time-to-Total Hip Arthroplasty

Factor	IRR (95% CI)	*P* value	TV *P* value
Race and ethnicity			
American Indian and Alaska Native	0.87 (0.66-1.14)	.30	.76
Asian and Pacific Islander	0.76 (0.66-0.88)	<.001	.63
Black	0.79 (0.74-0.85)	<.001	.002
Hispanic	0.84 (0.73-0.96)	.01	.11
White	1 [Reference]	NA	.45
Another Race and ethnicity	0.88 (0.77-1.00)	.06	.85
Beneficiary category			
Active-duty service member	1 [Reference]	NA	NA
Retired service member	1.39 (1.24-1.56)	<.001	>.99
Family member	1.48 (1.30-1.69)	<.001	.24
Sex			
Female	1 [Reference]	NA	NA
Male	1.20 (1.11-1.31)	<.001	.98
Age	Smooth^a^	<.001	.03
Diagnosis 1 y prior to index date			
Psychiatric diagnosis	0.79 (0.76-0.83)	<.001	NA
Pain-related diagnosis	0.69 (0.66-0.72)	<.001	NA
Obesity	0.92 (0.88-0.96)	<.001	NA
Nicotine dependence diagnosis or tobacco use	0.97 (0.91-1.04)	.36	NA
CCI category			
0	1 [Reference]	NA	.43
1	1.03 (0.91-1.17)	.61	.68
≥2	0.85 (0.74-0.97)	.02	>.99
Index System			
Direct care	1 [Reference]	NA	.002
Purchased care	0.38 (0.36-0.40)	<.001	<.001
Index diagnosis year	0.97 (0.95-0.99)	.001	.01
Preindex date injections			
0	1 [Reference]	NA	NA
1	0.83 (0.77-0.90)	<.001	NA
≥2	0.75 (0.70-0.81)	<.001	NA
Preindex date imaging			
0	1 [Reference]	NA	.99
1	0.93 (0.87-1.01)	.07	.10
≥2	0.77 (0.69-0.86)	<.001	<.001
Orthopedic surgeon visits	1.67 (1.64-1.69)	<.001	<.001
Opioid prescriptions	1.02 (1.01-1.03)	.002	<.001
Therapeutic visits	Smooth^a^	<.001	<.001
Imaging	1.26 (1.24-1.28)	<.001	.07
Geographic region			
East South Central	0.90 (0.81-1.00)	.05	.01
Midwest	1.35 (1.22-1.49)	<.001	.01
Mountain	1.34 (1.22-1.46)	<.001	.01
Northeast	0.96 (0.83-1.12)	.60	.01
Pacific	1.30 (1.20-1.40)	<.001	.02
South Atlantic	1 [Reference]	NA	NA
US Territory, another country, unknown	0.64 (0.52-0.78)	<.001	<.001
West South Central	0.96 (0.89-1.04)	.28	.02
Time, wk	Smooth^a^	<.001	NA

Abbreviations: CCI Charlson Comorbidity Index; IRR, incidence rate ratio; NA, not applicable; TV, time-varying.

^a^ When a TV *P* value is significant, the fixed or smooth covariate varies across time and may better approximate an average IRR across time (eFigures 2-5 in Supplement 1). Only variables with nonproportional hazards across each model had an additional time-varying covariate included.

IRRs and 3-year adjusted probabilities were lower for patients who had (probability, 21%; 95% CI, 27%-35%) vs did not have (probability, 37%; 95% CI, 32%-42%) a psychiatric diagnosis (IRR, 0.79, 0.76-0.83; *P* < .001), had (probability, 37%; 95% CI, 32%-42%) vs did not have (probability, 49%; 95% CI, 43%-55%) a pain-related diagnosis (IRR, 0.69; 95% CI, 0.66-0.72; *P* < .001), and had (probability, 34%; 95% CI, 30%-40%) vs did not have (probability, 37%, 32%-42%) an obesity diagnosis (IRR, 0.92; 95% CI, 0.88-0.96; *P* < .001) prior to the index diagnosis. Patients with a CCI score of 2 or greater were less likely to undergo THA (probability, 32%; 95% CI, 28%-36%) compared to those with a CCI score of 0 (probability, 35%; 95% CI, 30%-41%) (IRR, 0.85; 95% CI, 0.74-0.97; *P* = .02). Patients diagnosed in the purchased care system (probability, 18%; 95% CI, 16%-21%) compared with the direct care system (probability, 37%; 95% CI, 32%-42%) were less likely to undergo THA (IRR, 0.38; 95% CI, 0.36-0.40; *P* < .001), and those diagnosed in later years (eg, 2022: probability, 36%; 95% CI, 32%-42%) were less likely to receive THA than those diagnosed earlier (eg, 2018: probability, 38%; 95% CI, 33%-43%) (IRR, 0.97; 95% CI, 0.95-0.99, *P* = .001). Patients with 1 (probability, 32%; 95% CI, 27%-37%) or more (probability, 29%; 95% CI, 25%-34%) preindex injections were less likely to receive THA than those who received none (probability, 37%; 95% CI, 32%-42%) (1 injection: IRR, 0.83; 95% CI, 0.77-0.90; *P* < .001; >1 injections: IRR, 0.75; 95% CI, 0.70-0.81; *P* < .001). Patients with at least 2 preindex imaging visit days (probability, 34%; 95% CI, 28%-40%) were less likely to receive THA than those with 0 visit days (probability, 37%; 95% CI, 32%-42%) (IRR, 0.77; 95% CI, 0.69-0.86; *P* < .001) (Table 2; eFigure 2 in Supplement 1). Probabilities and incidences were higher for retired service members (probability, 49%; 95% CI, 45%-53%) and family members (probability, 50%; 95% CI, 45%-55%) compared with active-duty service members (probability, 37%; 95% CI, 32%-42%) (retired service members: IRR, 1.39; 95% CI, 1.24-1.56; *P* < .001; family members: IRR, 1.48; 95% CI, 1.30-1.69; *P* < .001). Male patients were more likely to receive THA (probability, 37%; 95% CI, 32%-42%) compared with female patients (probability, 32%; 95% CI, 28%-37%) (IRR, 1.20; 95% CI, 1.11-1.31; *P* < .001). Patients with increasing orthopedic surgeon visit days after the index diagnosis (eg, 0 days: probability, 22%; 95% CI, 19%-26%; vs 2 days: probability, 58%; 95% CI, 52%-64%) were more likely to receive THA (IRR, 1.67; 95% CI, 1.64-1.69; *P* < .001), as were those with more opioid prescriptions (eg, 0 prescriptions: probability, 37%; 95% CI, 32%-42%; vs 2 prescriptions: probability, 43%; 95% CI, 38%-49%; IRR, 1.02; 95% CI, 1.01-1.03; *P* = .002) or more imaging visit days (eg, 0 days: probability, 30%; 95% CI, 26%-35%; vs 2 days: probability, 45%; 95% CI, 39%-50%; IRR, 1.26; 95% CI, 1.24-1.28, *P* < .001) (Table 2; eFigure 2 in Supplement 1). IRRs varied in direction across geographic areas. Compared with patients diagnosed in the South Atlantic area (probability, 37%; 95% CI, 32%-42%), those diagnosed in the Midwest (probability, 46%; 95% CI, 40%-53%) (IRR, 1.35; 95% CI, 1.22-1.49; *P* < .001), Mountain (probability, 45%; 95% CI, 39%-51%) (IRR, 1.34; 95% CI, 1.22-1.46; *P* < .001), and Pacific (probability, 42%, 36%-48%) (IRR, 1.30; 95% CI, 1.20-1.40; *P* < .001) areas had higher incidence of THA; whereas, patients diagnosed in a US territory, another country, or an unknown location had lower incidence (probability, 24%; 95% CI, 18%-31%) (IRR, 0.64; 95% CI, 0.52-0.78; *P* < .001).

Several smooth and time-varying covariates were also significant, indicating that longitudinal hazards were nonproportional for several covariates (Table 2). Therefore, visualizations are provided in eFigures 2 to 5 in Supplement 1. Lastly, adjusted cumulative probabilities of THA receipt across covariate values at 1, 2, and 3 years after the index diagnosis are reported in eTable 4 in Supplement 1.

### Sensitivity Analysis

In the first set of sensitivity GAMs, 6896 patients were included in the direct care–only GAM and 20 821 patients were included in the purchased care–only GAM (eTable 2 in Supplement 1). Therefore, 9522 patients who had hip osteoarthritis care across both systems were removed from this analysis. There were 6165 patients included in the sensitivity analysis of patients receiving all hip osteoarthritis care in the direct care system with an index diagnosis at a military treatment facility with at least 25 included patients. Each sensitivity model required different time-varying covariates.

The significant associations found in the primary model were similar to the sensitivity models with some exceptions. Similar to the primary model, Black patients had lower IRRs across all 3 models (direct care: IRR, 0.74; 95% CI, 0.66-0.82; *P* < .001; purchased care: IRR, 0.77; 95% CI, 0.68-0.88; *P* < .001; direct care with facility random effect: IRR, 0.84; 95% CI, 0.73-0.96; *P* = .01) than White patients (eTable 3 and eFigure 4 in Supplement 1). Asian and Pacific Islander patients had lower IRR in both the primary and purchased care models (IRR, 0.72; 95% CI, 0.55-0.95; *P* = .02); there was a lack of statistically significant differences in the direct care models. While patients with another race and ethnicity did not significantly vary in time to THA in the primary model compared with White patients, the IRR was lower in the purchased care model (IRR, 0.80; 95% CI, 0.65-0.97; *P* = .02). The significantly lower IRRs in the primary analysis for Hispanic patients did not maintain in the sensitivity models. Male patients had higher IRRs in the primary and purchased care models (IRR, 1.29; 95% CI, 0.17-1.43; *P* < .001) compared with female patients, but not in the direct care sensitivity models. The association of having 2 or more preindex imaging visits with lower IRRs compared with none in the primary model was maintained in the purchased care model (IRR, 0.80; 95% CI, 0.68-0.93, *P* = .004) and direct care sensitivity model with the facility-level random effect (IRR, 0.76; 95% CI, 0.60-0.90; *P* = .02). Patients with CCI scores 2 or greater and fewer opioid prescriptions had lower IRRs in the primary model but lacked significance across the 3 sensitivity models.

IRR varied across geographic areas, with differences in the direction of associations across models. Compared with the South Atlantic area, patients in the Midwest had higher incidence of THA in the purchased care model (IRR, 1.28; 95% CI, 1.10-1.48; *P* = .001), similar to the primary model, but lower incidence in the direct care model with the facility-level random effect (IRR, 0.29; 95% CI, 0.12-1.70; *P* = .006). Similarly, patients in the Mountain area had higher incidence of THA in the purchased care model (IRR, 1.45; 95% CI, 1.27-1.66; *P* < .001), like the primary model, but lower incidence in the direct care model (IRR, 0.61; 95% CI, 0.46-0.80; *P* < .001).

## Discussion

In this cohort study, several patient-, care-, and structural-level factors were associated with time to THA in a sample of patients enrolled in TRICARE Prime, TRICARE Plus, and direct care–only plans. Similar to prior research, Asian and Pacific Islander, Black, and Hispanic patients, compared with White patients, and female patients, compared with male patients, were less likely to receive a THA across time.^6,7,8,13^ In sensitivity analyses of subsamples receiving all hip osteoarthritis care in the direct vs purchased care systems, Black patients and female patients had lower hazards of THA across time. While older patients and retired service members and family members had higher THA hazards relative to younger patients and active-duty service members, respectively, the discrepancies increased within the first year, then plateaued. Most athletes return to sports within 6 to 12 months of hip arthroplasty,^35^ but return may be contingent on the physical demands of the sport.^36^ For active-duty service members, not being able to maintain physical fitness requirements for an extended period may have negative career implications (eg, reduced deployability, medical separation).^37^ Alternatively, younger patients and active-duty service members may have received an osteoarthritis diagnosis earlier in the disease process, which could be more amenable to nonpharmacological and pharmacological management. Taken together, patient-level variation in time to THA within the MHS is similar to prior studies and warrants further investigation into osteoarthritis diagnosis timing and referral pathways.

Delay in THA may be due to systemic barriers across geographic areas, especially for patients in the purchased care system whose health care is not delivered in a centralized facility. In a 2018 study,^38^ Black patients had worse presurgical pain and functioning before THA; after surgery, Black patients residing in areas with higher proportions of Medicaid enrollees and people whose incomes were below the federal poverty threshold had worse outcomes than those living in less socioeconomically marginalized areas. However, White patients who resided in areas with high vs low socioeconomic marginalization lacked differences.^38^ In a Medicare claims evaluation,^39^ hospital service areas with higher proportions of Black residents tended to have fewer surgeons per capita. Other studies have reported that Black and Hispanic patients undergoing total joint arthroplasty were less likely to receive care from high-volume hospitals, which may result in worse outcomes.^40,41^ In this study, geographic area was associated with THA receipt, but variation across geographic areas with direct vs purchased care systems highlights the need to evaluate and incorporate orthopedic surgeon locations, structural factors, and beneficiary residences within geospatial modeling to optimize personnel allocation and care access.

Patients who received their index hip osteoarthritis diagnosis in purchased care were less likely to receive a THA across time relative to those diagnosed in direct care. This finding demonstrates challenges with purchased care access, which may be due to reduced reimbursement rates^42^ or increased care navigation demands. It is unclear whether increasing reimbursement rates or access to care navigators would improve THA timeliness. Prior research indicates that the Medicare Comprehensive Care for Joint Replacement Model may have improved total knee and hip arthroplasty accessibility for Hispanic patients but may have reduced accessibility for Black patients, with no changes in receipt for White patients.^43^ While increasing direct care recapture is a Department of Defense goal,^44^ it is unclear whether the direct care system has adequate capacity, per reports by both the Government Accountability Office and Department of Defense Inspector General.^45,46^ As a result, access to THA, as well as hip osteoarthritis care consistent with the policy-directed Stepped Care Model for Pain,^47^ may be stymied. Recommendations from these reports highlight the need to fully evaluate health care requirements, accessibility, barriers, and staffing to implement effective strategies across direct and purchased care systems.

Because the MHS is a single-payer system, surgeon allocation and care models could be altered to optimize timely THA access, reduce delay, and enhance value-based care. However, recent estimates indicate total joint arthroplasty volumes at military treatment facilities and among its orthopedic surgeons are generally low, and surgeons with the highest volume are still considered to have a relatively medium volume^48^ compared with prior research.^49^ Restructuring the MHS to enable high-volume arthroplasty hospitals and surgeons could improve patient outcomes and reduce costs.^40,41,50,51^ Future restructuring requires a thorough evaluation of factors preventing high-volume arthroplasty facilities and surgeons, as volume could be attributed to many factors (eg, surgical and imaging personnel, equipment, and room availability; patient transportation; and access to post-THA physical therapy).

### Limitations

This study has several limitations. Several factors associated with THA (eg, osteoarthritis symptom severity, hip joint trauma history, morphology, environmental exposures) were not available in these data, and the proportion of patients with posttraumatic hip osteoarthritis index diagnoses was small (1%). To account for symptom severity, analyses included time-dependent health care covariates that could offset THA need (eg, therapeutic visits) or made THA more likely (eg, imaging and orthopedic surgeon visits).^52^ The aggregated therapeutic visits variable incorporated many care types; specific conclusions regarding which therapies were attributed to THA timing were outside the study scope. Recorded diagnoses may be provisional and may not represent finalized or confirmed diagnoses. Therefore, there were several bivariate differences between patients who were included vs excluded from the sample due to not having a follow-up osteoarthritis visit at least 1 week after the index diagnosis. Evaluating why patients did not continue hip osteoarthritis care was outside the study scope. This study did not include patients whose race and ethnicity were not recorded in their records; bivariate analyses indicated that patients without race and ethnicity in their administrative records were disproportionately family members of TRICARE policy holders. Historically, TRICARE registration is done by the primary policy holder (eg, active-duty or retired service member), and policy holders are not required to report the race and ethnicity of family members. Therefore, findings may not generalize to patients who are family members. While management of hip and other lower-limb conditions could indirectly affect hip osteoarthritis symptoms and this study leveraged hip imaging and injection codes used in previous work, it is not possible to confirm their use was for hip osteoarthritis or the specific injection type. Due to the lack of adequate sample sizes at purchased care hospitals, analyses could not incorporate a random effect for facility in the primary and purchased care models. This study only included patients enrolled in specific TRICARE and direct care–only plans; therefore, generalizability to patients in other health care systems may be limited.

## Conclusions

This cohort study identified variation in time to THA among MHS beneficiaries. Consistent with US Government Accountability Office and Department of Defense Inspector General reports, future research could be used to improve THA accessibility and reduce barriers by accounting for structural inputs, such as personnel availability and reimbursement rates within the context of geographic location.

## Notes

Supplement 1. **eAppendix.** Diagnosis and procedure codes **eTable 1.** Sample descriptive information and bivariate analyses between patients who were included versus excluded due to missing race and ethnicity in administrative records and censoring within the first week **eTable 2.** Bivariate analyses between patients who received all hip osteoarthritis in the direct care system, purchased care system, and both systems **eTable 3.** Incidence rate ratios from the three sensitivity Poisson generalized additive models predicting time-to-total hip arthroplasty **eTable 4.** Covariate-adjusted cumulative probabilities of total hip arthroplasty receipt based on generalized additive model results **eFigure 1.** Unadjusted survival curves and censoring frequency table by race and ethnicity, sex, beneficiary group, age group, system of osteoarthritis diagnosis, and geographic area **eFigure 2.** Covariate-adjusted probabilities of total hip arthroplasty receipt. Note: Adjusted probabilities were based on a priori reference categories of non-focal variables **eFigure 3.** Cumulative hazard differences between comparator groups and reference groups for smooth and time-varying covariates in the primary model. **eFigure 4.** Cumulative hazard differences across race and ethnicity, relative to white patients across the primary and three sensitivity models. **eFigure 5.** Cumulative hazard differences across geographic region, relative to patients who received an index diagnosis in the South Atlantic region across the primary and three sensitivity models

Supplement 2.
Data Sharing Statement

## References

[1] Cooper GM, Bayram JM, Clement ND. The functional and psychological impact of delayed hip and knee arthroplasty: a systematic review and meta-analysis of 89,996 patients. Sci Rep. 2024;14(1):8032. doi:10.1038/s41598-024-58050-6 38580681 PMC10997604

[2] Singh JA, Yu S, Chen L, Cleveland JD. Rates of total joint replacement in the United States: future projections to 2020-2040 using the National Inpatient Sample. J Rheumatol. 2019;46(9):1134-1140. doi:10.3899/jrheum.170990 30988126

[3] Shichman I, Roof M, Askew N, . Projections and epidemiology of primary hip and knee arthroplasty in Medicare patients to 2040-2060. JB JS Open Access. 2023;8(1):e22.00112. doi:10.2106/JBJS.OA.22.00112 36864906 PMC9974080

[4] Qiu A, Meadows K, Ye F, Iyawe O, Kenneth-Nwosa K. Quantifying patient demand for orthopedics care by region through Google Trends analysis: descriptive epidemiology study. Online J Public Health Inform. 2025;17:e63560. doi:10.2196/63560 39888712 PMC11804898

[5] Sloan M, Premkumar A, Sheth NP. Future demand for total joint arthroplasty drives renewed interest in arthroplasty fellowship. HSS J. 2020;16(suppl 2):210-215. doi:10.1007/s11420-019-09678-y 33380948 PMC7749885

[6] Thirukumaran CP, Cai X, Glance LG, . Geographic variation and disparities in total joint replacement use for Medicare beneficiaries: 2009 to 2017. J Bone Joint Surg Am. 2020;102(24):2120-2128. doi:10.2106/JBJS.20.00246 33079898 PMC8190867

[7] Best MJ, McFarland EG, Thakkar SC, Srikumaran U. Racial disparities in the use of surgical procedures in the US. JAMA Surg. 2021;156(3):274-281. doi:10.1001/jamasurg.2020.6257 33439237 PMC7807389

[8] Hartnett DA, Brodeur PG, Kosinski LR, Cruz AI Jr, Gil JA, Cohen EM. Socioeconomic disparities in the utilization of total hip arthroplasty. J Arthroplasty. 2022;37(2):213-218.e1. doi:10.1016/j.arth.2021.10.021 34748913

[9] Hausmann LRM, Brandt CA, Carroll CM, . Racial and ethnic differences in total knee arthroplasty in the Veterans Affairs health care system, 2001-2013. Arthritis Care Res (Hoboken). 2017;69(8):1171-1178. doi:10.1002/acr.23137 27788302 PMC5538734

[10] Sowa H, Patzkowski J, Ismawan J, Velosky AG, Highland KB. Racialized inequities in knee arthroplasty receipt after osteoarthritis diagnosis in the US Military Health System. Arthritis Care Res (Hoboken). 2024;76(5):664-672. doi:10.1002/acr.25290 38185854

[11] Ezomo OT, Sun D, Gronbeck C, Harrington MA, Halawi MJ. Where do we stand today on racial and ethnic health disparities: an analysis of primary Total Hip Arthroplasty From a 2011-2017 National Database. Arthroplast Today. 2020;6(4):872-876. doi:10.1016/j.artd.2020.10.002 33163602 PMC7609456

[12] Cavanaugh AM, Rauh MJ, Thompson CA, . Racial and ethnic disparities in utilization of total knee arthroplasty among older women. Osteoarthritis Cartilage. 2019;27(12):1746-1754. doi:10.1016/j.joca.2019.07.015 31404657 PMC6875623

[13] Stronach BM, Zhang X, Haas D, Iorio R, Anoushiravani A, Barnes CL. Worsening arthroplasty utilization with widening racial variance during the COVID-19 pandemic. J Arthroplasty. 2022;37(7):1227-1232. doi:10.1016/j.arth.2022.03.001 35276272 PMC8904006

[14] Sabha M, Hochberg MC. Non-surgical management of hip and knee osteoarthritis; comparison of ACR/AF and OARSI 2019 and VA/DoD 2020 guidelines. Osteoarthr Cartil Open. 2021;4(1):100232. doi:10.1016/j.ocarto.2021.100232 36474466 PMC9718349

[15] Overton C, Nelson AE, Neogi T. Osteoarthritis treatment guidelines from six professional societies: similarities and differences. Rheum Dis Clin North Am. 2022;48(3):637-657. doi:10.1016/j.rdc.2022.03.009 35953228 PMC9377706

[16] Katz JN, Arant KR, Loeser RF. Diagnosis and treatment of hip and knee osteoarthritis: a review. JAMA. 2021;325(6):568-578. doi:10.1001/jama.2020.22171 33560326 PMC8225295

[17] Rees HW. Management of osteoarthritis of the hip. J Am Acad Orthop Surg. 2020;28(7):e288-e291. doi:10.5435/JAAOS-D-19-00416 31800436

[18] Kolasinski SL, Neogi T, Hochberg MC, . 2019 American College of Rheumatology/Arthritis Foundation guideline for the management of osteoarthritis of the hand, hip, and knee. Arthritis Rheumatol. 2020;72(2):220-233. doi:10.1002/art.41142 31908163 PMC10518852

[19] Veterans Administration/Department of Defense. The non-surgical management of hip & knee osteoarthritis (OA). Accessed June 22, 2024. https://www.healthquality.va.gov/guidelines/cd/oa/index.asp

[20] Karmarkar TD, Maurer A, Parks ML, . A fresh perspective on a familiar problem: examining disparities in knee osteoarthritis using a Markov model. Med Care. 2017;55(12):993-1000. doi:10.1097/MLR.0000000000000816 29036012 PMC5690313

[21] Abraham VM, Junge JM, Booth G, Olsen AA, Balazs GC, Goldman AH. Disparities in demographics in hip arthroplasty between U.S. active duty military and the ACS-NSQIP clinical registry. Mil Med. 2024;189(7-8):e1760-e1764. doi:10.1093/milmed/usae029 38345083

[22] Reif S, Adams RS, Ritter GA, Williams TV, Larson MJ. Prevalence of pain diagnoses and burden of pain among active duty soldiers, FY2012. Mil Med. 2018;183(9-10):e330-e337. doi:10.1093/milmed/usx200 29547946 PMC6115865

[23] Suissa K, Schneeweiss S, Lin KJ, Brill G, Kim SC, Patorno E. Validation of obesity-related diagnosis codes in claims data. Diabetes Obes Metab. 2021;23(12):2623-2631. doi:10.1111/dom.14512 34338404 PMC8578343

[24] Quan H, Li B, Couris CM, . Updating and validating the Charlson comorbidity index and score for risk adjustment in hospital discharge abstracts using data from 6 countries. Am J Epidemiol. 2011;173(6):676-682. doi:10.1093/aje/kwq433 21330339

[25] Wu M, Case A, Kim BI, . Racial and ethnic disparities in the imaging workup and treatment of knee and hip osteoarthritis. J Arthroplasty. 2022;37(8S):S753-S760, 760.e2. doi:10.1016/j.arth.2022.02.019 35151805

[26] US Census Bureau. Geographic levels. Updated October 8, 2021. Accessed August 18, 2025. https://www.census.gov/programs-surveys/economic-census/guidance-geographies/levels.html

[27] Subirana I, Sanz H, Vila J. Building bivariate tables: the Comparegroups package for R. J Stat Softw. 2014;57:1-16. doi:10.18637/jss.v057.i12 25400517

[28] Kassambara A, Kosinski M, Biecek P, Fabian S. Survminer: drawing survival curves using ‘ggplot2’. Accessed July 13, 2023. https://cran.r-project.org/web/packages/survminer/index.html

[29] Therneau T. A package for survival analysis in R. Accessed July 13, 2023. https://cran.r-project.org/web/packages/survival/index.html

[30] Bender A, Groll A, Scheipl F. A generalized additive model approach to time-to-event analysis. Stat Model. 2018;18(3-4):299-321. doi:10.1177/1471082X17748083

[31] Pedersen EJ, Miller DL, Simpson GL, Ross N. Hierarchical generalized additive models in ecology: an introduction with mgcv. PeerJ. 2019;7:e6876. doi:10.7717/peerj.6876 31179172 PMC6542350

[32] Ramjith J, Bender A, Roes KCB, Jonker MA. Recurrent events analysis with piece-wise exponential additive mixed models. Stat Model. 2024;24(3):266-287. doi:10.1177/1471082X221117612

[33] Lüdecke D. Sjplot: data visualization for statistics in social science. Accessed July 13, 2023. https://cran.r-project.org/web/packages/sjPlot/index.html

[34] Wickham H, Chang W. ggplot2: create elegant data visualisations using the grammar of graphics: version 3.5.1. Published April 23, 2024. Accessed March 5, 2025. https://cran.r-project.org/web/packages/ggplot2/index.html

[35] Magan AA, Radhakrishnan GT, Kayani B, . Time for return to sport following total hip arthroplasty: a meta-analysis. Hip Int. 2023;33(2):221-230. doi:10.1177/11207000211041975 34538122

[36] Sowers CB, Carrero AC, Cyrus JW, Ross JA, Golladay GJ, Patel NK. Return to sports after total hip arthroplasty: an umbrella review for consensus guidelines. Am J Sports Med. 2023;51(1):271-278. doi:10.1177/03635465211045698 34668788

[37] Walker SD, Abraham VM, Yancey I, Balazs GC, Goldman AH. Younger age is a risk factor for discontinuing military service after THA. Clin Orthop Relat Res. 2025. doi:10.1097/CORR.0000000000003581 40522258 PMC12517931

[38] Goodman SM, Mehta B, Zhang M, . Disparities in total hip arthroplasty outcomes: census tract data show interactions between race and community deprivation. J Am Acad Orthop Surg. 2018;26(21):e457-e464. doi:10.5435/JAAOS-D-17-00393 30192253

[39] Ghomrawi HMK, Funk RJ, Parks ML, Owen-Smith J, Hollingsworth JM. Physician referral patterns and racial disparities in total hip replacement: a network analysis approach. PLoS One. 2018;13(2):e0193014. doi:10.1371/journal.pone.0193014 29462180 PMC5819779

[40] Dy CJ, Marx RG, Ghomrawi HMK, Pan TJ, Westrich GH, Lyman S. The potential influence of regionalization strategies on delivery of care for elective total joint arthroplasty. J Arthroplasty. 2015;30(1):1-6. doi:10.1016/j.arth.2014.08.017 25282073

[41] Zalikha AK, Almsaddi T, Nham F, Hussein IH, El-Othmani MM. Comorbidity, racial, and socioeconomic disparities in total knee and hip arthroplasty at high versus low-volume centers. J Am Acad Orthop Surg. 2023;31(5):e264-e270. doi:10.5435/JAAOS-D-22-00665 36729540

[42] Anand P, Ben-Shalom Y, Schone E. Factors associated with the acceptance of new TRICARE and Medicare patients by health care providers. Med Care Res Rev. 2021;78(5):627-637. doi:10.1177/1077558720942700 32696719

[43] Kim H, Meath THA, Quiñones AR, McConnell KJ, Ibrahim SA. Association of Medicare mandatory bundled payment program with the receipt of elective hip and knee replacement in White, Black, and Hispanic beneficiaries. JAMA Netw Open. 2021;4(3):e211772. doi:10.1001/jamanetworkopen.2021.1772 33749766 PMC7985721

[44] Military MR. Health System stabilization: rebuilding health care access is critical to patient's well-being. News release. US Department of War. January 22, 2024. Accessed September 26, 2025. https://www.war.gov/News/News-Stories/Article/article/3652092/military-health-system-stabilization-rebuilding-health-care-access-is-critical/

[45] US Government Accountability Office. Military installations: DoD should consider various support services when designating sites as remote or isolated. July 29, 2021. Accessed September 24, 2025. https://www.gao.gov/products/gao-21-276

[46] Management advisory: concerns with access to care and staffing shortages in the Military Health System. Published November 29, 2023. Accessed September 24, 2025. https://www.dodig.mil/reports.html/Article/3602650/management-advisory-concerns-with-access-to-care-and-staffing-shortages-in-the/

[47] Defense Health Agency. Pain management and opioid safety in the Military Health System (MHS). Published July 8, 2018. Accessed March 5, 2025. https://www.health.mil/-/media/Files/MHS/Policy-Files/SIGNED--DHAPI-602504-Pain-Management-and-Opioid-Safety-in-the-MHS.ashx

[48] Formby PM, Rodkey DL. Most military arthroplasty surgeons have a low volume practice in the Military Health System. Arthroplast Today. 2024;25:101295. doi:10.1016/j.artd.2023.101295 38380159 PMC10877328

[49] Siddiqi A, Alamanda VK, Barrington JW, . Effects of hospital and surgeon volume on patient outcomes after total joint arthroplasty: reported from the American Joint Replacement Registry. J Am Acad Orthop Surg. 2022;30(11):e811-e821. doi:10.5435/JAAOS-D-21-00946 35191864

[50] Mufarrih SH, Ghani MOA, Martins RS, . Effect of hospital volume on outcomes of total hip arthroplasty: a systematic review and meta-analysis. J Orthop Surg Res. 2019;14(1):468. doi:10.1186/s13018-019-1531-0 31881918 PMC6935169

[51] Koltsov JCB, Marx RG, Bachner E, McLawhorn AS, Lyman S. Risk-based hospital and surgeon-volume categories for total hip arthroplasty. J Bone Joint Surg Am. 2018;100(14):1203-1208. doi:10.2106/JBJS.17.00967 30020125

[52] Amen TB, Varady NH, Wright-Chisem J, Bovonratwet P, Parks ML, Ast MP. Emerging racial disparities in outpatient utilization of total joint arthroplasty. J Arthroplasty. 2022;37(11):2116-2121. doi:10.1016/j.arth.2022.05.008 35537609

