# Supplementary material for: Time to Total Hip Arthroplasty Among Patients in the US Military Health System
Source: JAMA Netw Open. 2025 Oct 28;8(10):e2539971. doi: 10.1001/jamanetworkopen.2025.39971 (PMC12569716; doi:10.1001/jamanetworkopen.2025.39971)
Supplement: Supplement 1. — eAppendix. Diagnosis and procedure codes eTable 1. Sample descriptive information and bivariate analyses between patients who were included versus excluded due to missing race and ethnicity in administrative records and censoring within the first week eTable 2. Bivariate analyses between patients who received all hip osteoarthritis in the direct care system, purchased care system, and both systems eTable 3. Incidence rate ratios from the three sensitivity Poisson generalized additive models predicting time-to-total hip arthroplasty eTable 4. Covariate-adjusted cumulative probabilities of total hip arthroplasty receipt based on generalized additive model results eFigure 1. Unadjusted survival curves and censoring frequency table by race and ethnicity, sex, beneficiary group, age group, system of osteoarthritis diagnosis, and geographic area eFigure 2. Covariate-adjusted probabilities of total hip arthroplasty receipt. Note: Adjusted probabilities were based on a priori reference categories of non-focal variables eFigure 3. Cumulative hazard differences between comparator groups and reference groups for smooth and time-varying covariates in the primary model. eFigure 4. Cumulative hazard differences across race and ethnicity, relative to white patients across the primary and three sensitivity models. eFigure 5. Cumulative hazard differences across geographic region, relative to patients who received an index diagnosis in the South Atlantic region across the primary and three sensitivity models [file jamanetwopen-e2539971-s001.pdf]

Supplemental Online Content

Hillery BL, Goldman AH, Velosky AG, Amoako MY, Leggit JC, Highland KB. Time to total hip arthroplasty among patients in the US Military Health System. *JAMA Netw Open*. 2025;8(10):e2539971. doi:10.1001/jamanetworkopen.2025.39971

eAppendix. Diagnosis and procedure codes

eTable 1. Sample descriptive information and bivariate analyses between patients who were included versus excluded due to missing race and ethnicity in administrative records and censoring within the first week

eTable 2. Bivariate analyses between patients who received all hip osteoarthritis in the direct care system, purchased care system, and both systems

eTable 3. Incidence rate ratios from the three sensitivity Poisson generalized additive models predicting time-to-total hip arthroplasty

eTable 4. Covariate-adjusted cumulative probabilities of total hip arthroplasty receipt based on generalized additive model results

eFigure 1. Unadjusted survival curves and censoring frequency table by race and ethnicity, sex, beneficiary group, age group, system of osteoarthritis diagnosis, and geographic area

eFigure 2. Covariate-adjusted probabilities of total hip arthroplasty receipt. Note: Adjusted probabilities were based on a priori reference categories of non-focal variables

eFigure 3. Cumulative hazard differences between comparator groups and reference groups for smooth and time-varying covariates in the primary model

eFigure 4. Cumulative hazard differences across race and ethnicity, relative to white patients across the primary and three sensitivity models

eFigure 5. Cumulative hazard differences across geographic region, relative to patients who received an index diagnosis in the South Atlantic region across the primary and three sensitivity models

This supplemental material has been provided by the authors to give readers additional information about their work.

© 2025 Hillery B et al. *JAMA Network Open*.

eAppendix. Diagnosis and Procedure Codes

Abbreviations: CPT = Current Procedural Terminology, ICD-10 = International Classification of Diseases (Diagnoses), ICD-10 PCS = International Classification of Diseases Procedure Coding System

Hip osteoarthritis: ICD-10 M16, M16.0 (bilateral primary), 16.* (unilateral primary), 16.2 (bilateral from hip dysplasia), 16.3* (unilateral from hip dysplasia), 16.4 (bilateral post-traumatic), 16.5* (unilateral post-traumatic), 16.6 (other bilateral), 16.7 (other unilateral), 16.9 (unspecified); ICD-9 715.15 (primary), 715.25 (secondary), 715.35 (unspecified), 715.95 (unspecified)

Hip arthroplasty: CPT 27130; ICD-10 PCS 0SR9*, 0SRA*, 0SRB*, 0SRE*, 0SRR*, 0SRS*

Revision or conversion hip arthroplasty: CPT 27132, 27134, 27137, 27138; ICD-10 PCS 0SW*

Hip imaging (CPT)
- MRI: 73721, 73722, 73723, 72195, 72196, 72197
- CT: 73700, 73701, 73702
- X-Ray: 73501, 73502, 73503, 73523, 73522, 73521, 72170, 73500, 73510, 73520, 73540, 73550, 73525

Hip injections (CPT): 20610 (arthrocentesis, aspiration and/or injection, major joint or bursa without ultrasound), 20611 (arthrocentesis, aspiration and/or injection, major joint or bursa with ultrasound), 77002 (injection with fluoroscopic guidance)

Exclusion criteria (ICD-10):
- **Cancer**: C40.2, C40.20, C40.21, C40.22, C40.3, C40.30, C40.31, C40.32, C40.8, C40.80, C40.81, C40.82, C40.9, C40.90, C40.91, C40.92
- **Infection**: M00, M00.0, M00.00, M00.051, M00.052, M00.059, M00.06, M00.061, M00.062, M00.069, M00.09, M00.1, M00.10, M00.15, M00.151, M00.152, M00.159, M00.16, M00.161, M00.162, M00.169, M00.19, M00.2, M00.20, M00.25, M00.251, M00.252, M00.259, M00.26, M00.261, M00.262, M00.269, M00.29, M00.8, M00.80, M00.85, M00.851, M00.852, M00.859, M00.86, M00.861, M00.862, M00.869, M00.89, M00.9, M01.X5, M01.X51, M01.X52, M01.X59, M01.X6, M01.X61, M01.X62, M01.X69, M01.X9
- **Fracture**: S72.0, S72.1, S72.2, S72.3, S72.4, S72.8, S72.9, S82.0, S82.1, S82.2, S82.3, S82.4, S82.5, S82.6, S82.8, S82.9

Note: many codes are derived from Wu M, Case A, Kim BI, et al. Racial and Ethnic Disparities in the Imaging Workup and Treatment of Knee and Hip Osteoarthritis. The Journal of Arthroplasty. 2022;37(8):S753-S760.e2. doi:10.1016/j.arth.2022.02.019

© 2025 Hillery B et al. *JAMA Network Open*.

eTable 1. Sample descriptive information and bivariate analyses between patients who were included versus excluded due to (a) missing race and ethnicity in administrative records and (b) censoring within the first week.

	Final Analytic Sample (N = 37,239)	Missing Race & Ethnicity (a) (n = 211,337)	Censored at Week 1 (b) (n = 145,790)
Age	59 [50;64]	75 [67;81]	68 [59;74]
Beneficiary Category			
Active Duty	5730 (15%)	12 (0%)	6596 (5%)
Family Member	13229 (36%)	174699 (83%)	27753 (19%)
Guard	0 (0%)	21 (0%)	6617 (5%)
Other	0 (0%)	279 (0%)	682 (0%)
Retiree	18280 (49%)	35994 (17%)	103580 (71%)
Unknown	0 (0%)	332 (0%)	562 (0%)
Sex			
Female	15686 (42%)	173458 (82%)	38768 (27%)
Male	21553 (58%)	37879 (18%)	107022 (73%)
Index System			
Direct	13120 (35%)	628 (0%)	13732 (9%)
Purchased	24119 (65%)	210709 (100%)	132058 (91%)
Index Year	2021 [2019;2022]	2021 [2019;2022]	2021 [2019;2023]
Enrollment Group			
Direct	199 (1%)	134 (0%)	625 (0%)
Other	0 (0%)	171430 (81%)	86060 (59%)
Plus	8494 (23%)	3561 (2%)	7038 (5%)
Prime	28546 (77%)	14749 (7%)	31384 (22%)
Select	0 (0%)	21463 (10%)	20683 (14%)
Geographic Area			
East South Central	2601 (7%)	18607 (9%)	12821 (9%)
Midwest	2873 (8%)	32221 (15%)	20851 (14%)
Mountain	3489 (9%)	21914 (10%)	14346 (10%)
Northeast (Middle Atlantic, New England)	1196 (3%)	16551 (8%)	10268 (7%)
Pacific	4830 (13%)	22613 (11%)	14733 (10%)
South Atlantic	15059 (40%)	65686 (31%)	49513 (34%)
US Territory, Another Country, Unknown	947 (3%)	6138 (3%)	3357 (2%)
West South Central	6244 (17%)	27607 (13%)	19901 (14%)
Race and Ethnicity			
American Indian & Alaska Native	320 (1%)		1325 (1%)
Asian & Pacific Islander	1603 (4%)		4506 (3%)
Black	8123 (22%)		19931 (14%)
Latine	2041 (5%)		5173 (4%)
Other	1825 (5%)		6538 (4%)
White	23327 (63%)		108317 (74%)

Note: Values displayed are medians [interquartile ranges] and frequencies (percentages). P-values correspond to non-parametric bivariate tests. All median difference tests and chi-square analyses were statistically significant ($p < 0.001$). Some cell sizes in the final sample are 0 due to application of other exclusion criteria.

© 2025 Hillery B et al. *JAMA Network Open*.

eTable 2. Bivariate analyses with pairwise comparisons between patients who received all hip osteoarthritis in the direct care system (D), purchased care system (P), and both systems (B).

	**Direct Care Only (D)**	**Both Direct and Purchased Care (B)**	**Purchased Care Only (P)**	**DvB p**	**DvP p**	**BvP p**
	n = 6896	n = 9522	n = 20821			
Race and Ethnicity				<0.001	<0.001	<0.001
American Indian & Alaska Native	58 (1%)	73 (1%)	189 (1%)			
Asian & Pacific Islander	405 (6%)	426 (4%)	772 (4%)			
Black	1745 (25%)	2177 (23%)	4201 (20%)			
Hispanic	466 (7%)	524 (6%)	1051 (5%)			
Another Race & Ethnicity	305 (4%)	446 (5%)	1074 (5%)			
White	3917 (57%)	5876 (62%)	13534 (65%)			
Beneficiary Group				<0.001	<0.001	<0.001
Active Duty Service Member	1964 (28%)	1723 (18%)	2043 (10%)			
Family Member	2313 (34%)	3775 (40%)	7141 (34%)			
Retired Service Member	2619 (38%)	4024 (42%)	11637 (56%)			
Sex				<0.001	0.85	<0.001
Female	2847 (41%)	4272 (45%)	8567 (41%)			
Male	4049 (59%)	5250 (55%)	12254 (59%)			
Age (years)	58 [47;65]	59 [50;65]	59 [51;64]	<0.001	<0.001	0.49
Age-Adjusted CCI Category				<0.001	<0.001	<0.001
0	1760 (26%)	1890 (20%)	3577 (17%)			
1	1480 (21%)	2109 (22%)	4562 (22%)			
2+	3656 (53%)	5523 (58%)	12682 (61%)			
1-Year Pre-Index Psychiatric Diagnosis	1711 (25%)	2329 (24%)	5917 (28%)	0.62	<0.001	<0.001
1-Year Pre-Index Pain-Related Diagnosis	5696 (83%)	7839 (82%)	17750 (85%)	0.66	<0.001	<0.001
1-Year Pre-Index Obesity Diagnosis	1261 (18%)	1955 (21%)	5359 (26%)	<0.001	<0.001	<0.001
1-Year Nicotine Dependence Diagnosis or Doc. Tobacco Use	954 (14%)	1201 (13%)	2122 (10%)	0.02	<0.001	<0.001
3-Year Pre-Index Hip Injections				0.06	<0.001	0.03
0	5751 (83%)	8058 (85%)	17874 (86%)			
1	511 (7%)	686 (7%)	1402 (7%)			
2+	634 (9%)	778 (8%)	1545 (7%)			
3-Year Pre-Index Hip Imaging				<0.001	<0.001	<0.001
0	5540 (80%)	7404 (78%)	15141 (73%)			
1	930 (13%)	1509 (16%)	3653 (18%)			
2+	426 (6%)	609 (6%)	2027 (10%)			
Index System:				<0.001	<0.001	<0.001
Direct	6896 (100%)	6224 (65%)	0 (0%)			
Purchased	0 (0%)	3298 (35%)	20821 (100%)			
Index Year	2021 [2019;2022]	2020 [2019;2022]	2021 [2019;2022]	0.05	<0.001	<0.001
Geographic Area				<0.001	<0.001	<0.001
East South Central	276 (4%)	610 (6%)	1715 (8%)			
Midwest	423 (6%)	654 (7%)	1796 (9%)			
Mountain	510 (7%)	831 (9%)	2148 (10%)			
Northeast	89 (1%)	235 (2%)	872 (4%)			
Pacific	1028 (15%)	1421 (15%)	2381 (11%)			
South Atlantic	2892 (42%)	3990 (42%)	8177 (39%)			
US Territory, Another Country, Unknown	414 (6%)	320 (3%)	213 (1%)			
West South Central	1264 (18%)	1461 (15%)	3519 (17%)			

Note: CCI = age-adjusted Charlson Comorbidity Index scores, Doc. = documented. Values displayed are medians [interquartile ranges] and frequencies (percentages). P-values correspond to non-parametric bivariate tests. Pairwise p-values are adjusted using the Benjamini and Hochberg method.

© 2025 Hillery B et al. *JAMA Network Open*.

eTable 3. Incidence rate ratios (IRR) from the three sensitivity Poisson generalized additive models predicting time-to-total hip arthroplasty.

	Purchased Care Only (n = 17721)				Direct Care Only (n = 6823)				Direct Care Only with Facility Random Effect (n = 6106)			
**Predictors**	**IRR**	**95% CI**	**p**	**TV p**	**IRR**	**95% CI**	**p**	**TV p**	**IRR**	**95% CI**	**p**	**TV p**
Race & Ethnicity (Ref. White)				0.28								
American Indian & Alaska Native	0.90	0.61-1.34	0.61	0.07	1.18	0.65-2.14	0.60		1.04	0.55-1.97	0.90	
Another Race & Ethnicity	0.80	0.65-0.97	**0.02**	0.89	1.22	0.95-1.57	0.12		1.27	0.98-1.64	0.07	
Asian & Pacific Islander	0.72	0.55-0.95	**0.02**	0.30	0.86	0.67-1.11	0.26		0.94	0.72-1.24	0.68	
Black	0.74	0.66-0.82	**<0.001**	0.07	0.77	0.68-0.88	**<0.001**		0.84	0.73-0.96	**0.01**	
Hispanic	0.81	0.66-1.00	0.05	0.68	1.04	0.83-1.31	0.73		1.08	0.84-1.37	0.56	
Beneficiary Category (Ref. Active Duty)								0.53				0.99
Retired Service Members	1.68	1.43-1.97	**<0.001**		1.97	1.49-2.60	**<0.001**	0.98	2.17	1.60-2.95	**<0.001**	1.00
Family Members	1.73	1.45-2.06	**<0.001**		2.01	1.50-2.71	**<0.001**	**0.02**	2.06	1.49-2.85	**<0.001**	**0.02**
Sex (Ref. Female)												
Male	1.29	1.17-1.43	**<0.001**		0.92	0.80-1.07	0.30		0.90	0.77-1.04	0.16	
Age	Smooth		**<0.001**	**0.01**	Smooth		**<0.001**		Smooth		**<0.001**	
Pre-Index Psychiatric Diagnosis (Ref. No)												
Yes	0.82	0.77-0.88	**<0.001**		0.67	0.59-0.77	**<0.001**		0.68	0.59-0.78	**<0.001**	
Pre-Index Pain-Related Diagnosis (Ref. No)												
Yes	0.67	0.63-0.72	**<0.001**		0.72	0.64-0.81	**<0.001**		0.78	0.69-0.89	**<0.001**	
Pre-Index Obesity Diagnosis (Ref. No)												
Yes	0.93	0.87-0.99	**0.02**		0.78	0.69-0.90	**<0.001**		0.84	0.73-0.96	**0.01**	
Pre-Index Nicotine Dependence Diagnosis or Tobacco Use (Ref. No)												
Yes	1.01	0.91-1.11	0.89		0.93	0.79-1.08	0.33		1.04	0.89-1.23	0.61	
CCI Category (Ref. 0)				1.00								
1	1.10	0.92-1.31	0.32	0.22	0.84	0.63-1.13	0.25		0.91	0.67-1.24	0.56	
2+	0.84	0.69-1.03	0.10	1.00	0.72	0.52-1.00	0.05		0.79	0.56-1.12	0.19	
Pre-Index Injections (Ref. 0)				0.98								
1	0.94	0.80-1.09	0.39	0.21	0.69	0.56-0.85	**<0.001**		0.74	0.60-0.91	**0.005**	
2+	0.86	0.74-0.99	**0.04**	0.30	0.73	0.61-0.88	**0.001**		0.77	0.64-0.93	**0.006**	
Pre-Index Imaging (Ref. 0)				0.99								
1	0.99	0.90-1.10	0.91	0.10	1.02	0.87-1.19	0.85		1.02	0.87-1.20	0.81	
2+	0.80	0.68-0.93	**0.004**	**<0.001**	0.86	0.68-1.08	0.19		0.76	0.60-0.96	**0.02**	
Orthopedic Surgeon Visits	1.47	1.44-1.50	**<0.001**	**<0.001**	2.54	2.44-2.64	**<0.001**	**<0.001**	2.62	2.51-2.74	**<0.001**	**<0.001**
Therapeutic Visits	Smooth		**<0.001**	**<0.001**	Smooth		**<0.001**	**<0.001**	Smooth		**<0.001**	**<0.001**
Imaging	1.30	1.27-1.33	**<0.001**	**0.04**	1.24	1.16-1.32	**<0.001**	**<0.001**	1.17	1.09-1.25	**<0.001**	**<0.001**

© 2025 Hillery B et al. *JAMA Network Open*.

Opioid Prescriptions	1.00	0.99-1.01	0.91	**<0.001**	1.03	1.00-1.07	0.09	**<0.001**	1.03	1.00-1.07	0.08	**0.001**
Index Diagnosis Year	1.03	1.01-1.05	**<0.001**		0.92	0.89-0.95	**<0.001**		0.91	0.88-0.95	**<0.001**	
Geographic Area (Ref. South Atlantic)				**0.001**				0.86				0.77
East South Central	1.10	0.96-1.27	0.16	**0.006**	0.74	0.47-1.18	0.21	0.84	0.99	0.50-1.97	0.98	0.89
Midwest	1.28	1.10-1.48	**0.001**	**0.001**	0.74	0.52-1.07	0.11	0.88	0.29	0.12-0.70	**0.006**	0.90
Mountain	1.45	1.27-1.66	**<0.001**	**0.001**	0.61	0.46-0.80	**<0.001**	0.70	0.71	0.40-1.26	0.24	0.72
Northeast	1.06	0.87-1.30	0.56	**0.001**	0.23	0.04-1.21	0.08	0.56	0.47	0.08-2.67	0.40	0.75
Pacific	1.37	1.21-1.55	**<0.001**	**0.009**	1.09	0.88-1.34	0.43	**0.001**	1.06	0.69-1.61	0.79	**0.03**
US Territory, Another Country, Unknown	0.70	0.43-1.15	0.16	**0.003**	0.60	0.40-0.90	**0.01**	0.42	0.65	0.34-1.25	0.20	0.58
West South Central	1.02	0.91-1.15	0.71	**0.001**	0.73	0.58-0.91	**0.005**	0.94	0.81	0.52-1.26	0.35	0.93
Time (Weeks)	Smooth		**<0.001**		Smooth		**<0.001**		Smooth		**<0.001**	
Facility Random Effect									Smooth		**<0.001**	

Note: TV p = time varying covariate p-value. If the TV p is significant, then the fixed or smooth covariate varies across time and may better approximate an average IRR, than a constant IRR across time. If smooth or TV variables are significant, use figures to facilitate interpretation. Only variables with nonproportional hazards across each model had an additional time-varying covariate included.

© 2025 Hillery B et al. *JAMA Network Open*.

eTable 4. Covariate-adjusted cumulative probabilities of total hip arthroplasty receipt based on generalized additive model results

Covariate	Value	Year 1 Estimate (95% CI)	Year 2 Estimate (95% CI)	Year 3 Estimate (95% CI)
Race and Ethnicity	American Indian & Alaska Native	24% (18%, 32%)	29% (22%, 38%)	32% (24%, 42%)
	Another Race & Ethnicity	25% (21%, 30%)	30% (25%, 35%)	33% (28%, 39%)
	Asian & Pacific Islander	23% (20%, 28%)	28% (23%, 33%)	30% (25%, 36%)
	Black	21% (18%, 24%)	26% (22%, 29%)	28% (24%, 33%)
	Hispanic	27% (23%, 32%)	32% (27%, 37%)	34% (29%, 40%)
	White	29% (25%, 33%)	34% (30%, 38%)	37% (32%, 42%)
Age	30 Years	10% (8%, 12%)	12% (10%, 15%)	13% (11%, 17%)
	50 Years	21% (19%, 24%)	26% (22%, 29%)	28% (24%, 32%)
	70 Years	32% (28%, 37%)	38% (33%, 43%)	41% (35%, 47%)
Beneficiary Category	Active Duty	29% (25%, 33%)	34% (30%, 38%)	37% (32%, 42%)
	Retiree	40% (37%, 43%)	46% (42%, 50%)	49% (45%, 53%)
	Family Member	40% (36%, 44%)	46% (42%, 51%)	50% (45%, 55%)
Sex	Female	25% (21%, 29%)	29% (25%, 34%)	32% (28%, 37%)
	Male	29% (25%, 33%)	34% (30%, 38%)	37% (32%, 42%)
Charlson Comorbidity Index Score	0	27% (23%, 31%)	32% (27%, 37%)	35% (30%, 41%)
	1	29% (25%, 33%)	34% (30%, 38%)	37% (32%, 42%)
	2+	25% (22%, 28%)	29% (26%, 33%)	32% (28%, 36%)
Index System	Direct	29% (25%, 33%)	34% (30%, 38%)	37% (32%, 42%)
	Purchased	15% (13%, 17%)	17% (15%, 20%)	18% (16%, 21%)
Index Year	2018	29% (25%, 33%)	34% (30%, 39%)	38% (33%, 43%)
	2020	29% (25%, 33%)	34% (30%, 39%)	37% (32%, 42%)
	2022	29% (25%, 33%)	34% (30%, 38%)	36% (32%, 42%)
Psychiatric Diagnosis	No	29% (25%, 33%)	34% (30%, 38%)	37% (32%, 42%)
	Yes	24% (20%, 27%)	28% (24%, 32%)	31% (27%, 35%)
Pain-Related Diagnosis	No	39% (34%, 44%)	45% (40%, 51%)	49% (43%, 55%)
	Yes	29% (25%, 33%)	34% (30%, 38%)	37% (32%, 42%)
Obesity Diagnosis	No	29% (25%, 33%)	34% (30%, 38%)	37% (32%, 42%)
	Yes	27% (23%, 31%)	32% (28%, 36%)	34% (30%, 40%)
Nicotine Dependence/Tobacco Use	No	29% (25%, 33%)	34% (30%, 38%)	37% (32%, 42%)
	Yes	28% (24%, 32%)	33% (29%, 38%)	36% (31%, 41%)
Pre-Index Imaging	0	29% (25%, 33%)	34% (30%, 38%)	37% (32%, 42%)
	1	28% (25%, 33%)	33% (29%, 38%)	36% (31%, 42%)
	2+	27% (23%, 32%)	31% (27%, 37%)	34% (28%, 40%)
Pre-Index Injections	0	29% (25%, 33%)	34% (30%, 38%)	37% (32%, 42%)
	1	25% (21%, 28%)	29% (25%, 34%)	32% (27%, 37%)
	2+	23% (19%, 26%)	27% (23%, 31%)	29% (25%, 34%)
Therapeutic Visits	0	12% (10%, 14%)	14% (12%, 17%)	15% (13%, 18%)
	4	47% (42%, 52%)	56% (50%, 61%)	60% (54%, 67%)
	8	38% (34%, 44%)	51% (45%, 57%)	57% (51%, 64%)
Imaging Visits	0	23% (20%, 26%)	28% (24%, 31%)	30% (26%, 35%)
	1	29% (25%, 33%)	34% (30%, 38%)	37% (32%, 42%)
	2	35% (31%, 40%)	41% (36%, 47%)	45% (39%, 50%)
Opioid Prescriptions	0	29% (25%, 33%)	34% (30%, 38%)	37% (32%, 42%)
	1	32% (28%, 37%)	37% (33%, 42%)	40% (35%, 45%)
	2	36% (32%, 41%)	41% (36%, 46%)	43% (38%, 49%)
Orthopedic Surgeon Visits	0	16% (14%, 18%)	20% (17%, 23%)	22% (19%, 26%)
	1	29% (25%, 33%)	34% (30%, 38%)	37% (32%, 42%)
	2	49% (43%, 54%)	55% (49%, 60%)	58% (52%, 64%)
Geographic Area	South Atlantic	29% (25%, 33%)	34% (30%, 38%)	37% (32%, 42%)
	East South Central	27% (23%, 32%)	31% (27%, 37%)	34% (29%, 41%)
	Midwest	37% (32%, 42%)	43% (38%, 49%)	46% (40%, 53%)

© 2025 Hillery B et al. *JAMA Network Open*.

Mountain	35% (30%, 40%)	41% (36%, 47%)	45% (39%, 51%)
Northeast	29% (24%, 34%)	34% (28%, 40%)	37% (30%, 44%)
US Territory, Another Country, Unknown	17% (13%, 22%)	21% (16%, 28%)	24% (18%, 31%)
Pacific	32% (28%, 36%)	38% (33%, 44%)	42% (36%, 48%)
West South Central	28% (24%, 32%)	33% (29%, 38%)	36% (31%, 41%)

Note: Cumulative probabilities were adjusted with the following applicable assumptions for non-vocal variables: age = 57.2 years, beneficiary category = retired service member, index diagnosis system = purchased care, index diagnosis year = 2020.3, sex = male, race and ethnicity = white, psychiatric diagnosis = no, other pain diagnosis = yes, obesity diagnosis = no, nicotine diagnosis/tobacco use = no, pre-imaging and injections = 0, therapeutic visits = 4, imaging visits = 1, opioid prescriptions = 1.5, orthopedic surgeon visits = 0.64, geographic area = South Atlantic.

© 2025 Hillery B et al. *JAMA Network Open*.

eFigure 1.a. Unadjusted survival curves and censoring frequency table by race and ethnicity.

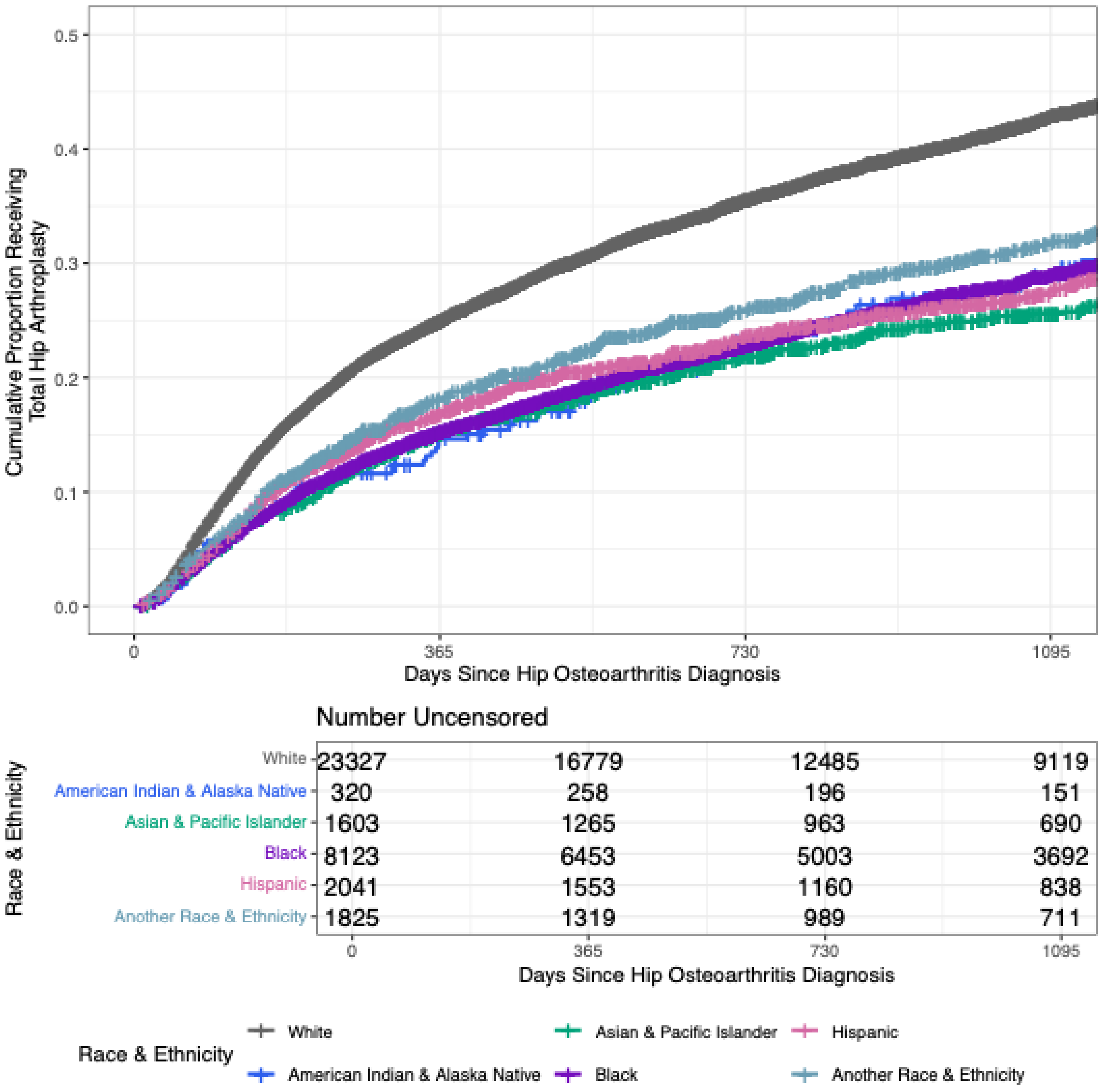

© 2025 Hillery B et al. *JAMA Network Open*.

eFigure 1.b. Unadjusted survival curves and censoring frequency table by sex.

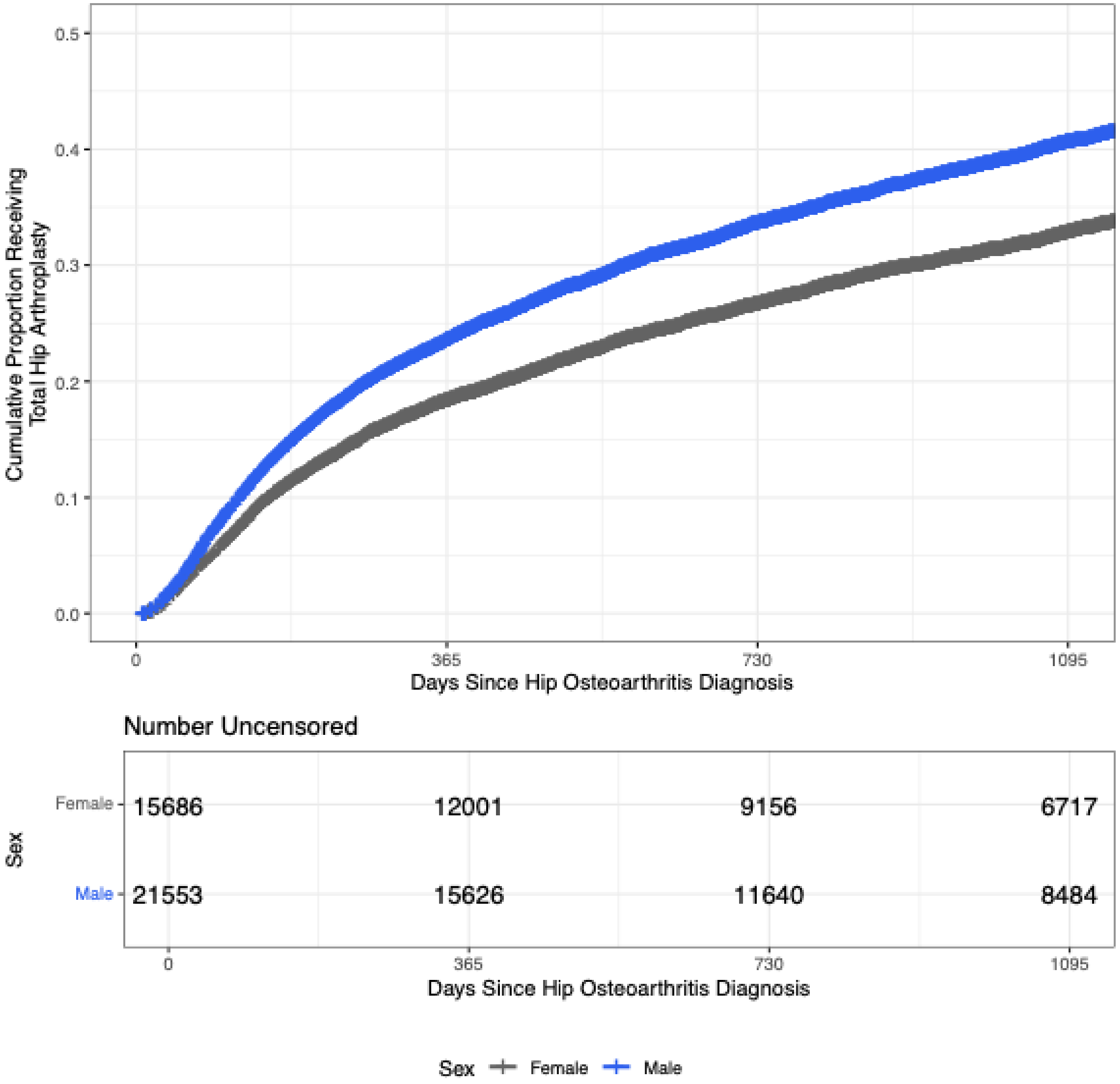

© 2025 Hillery B et al. *JAMA Network Open*.

eFigure 1.c. Unadjusted survival curves and censoring frequency table by beneficiary group.

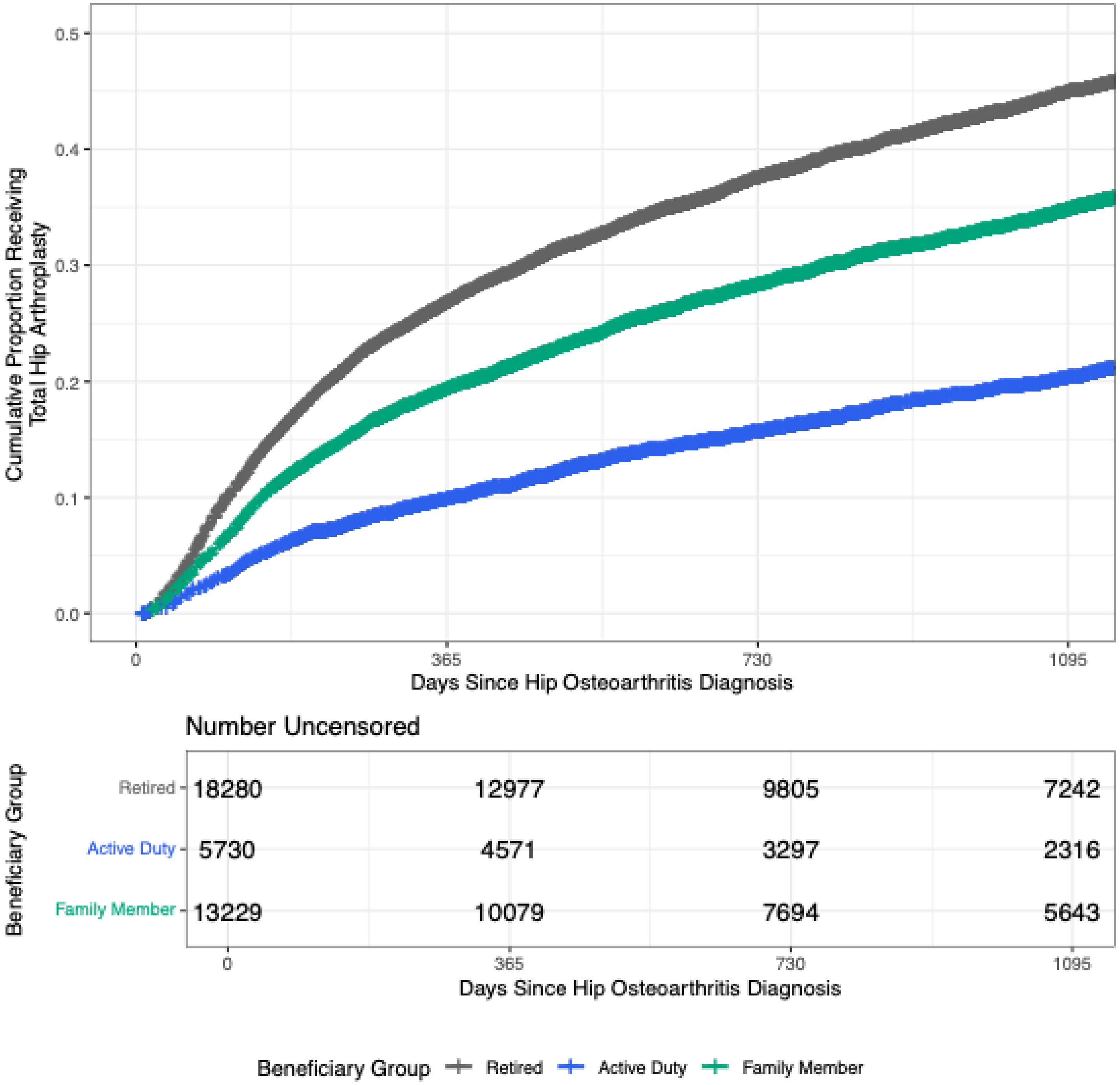

© 2025 Hillery B et al. *JAMA Network Open*.

eFigure 1.d. Unadjusted survival curves and censoring frequency table by age group.

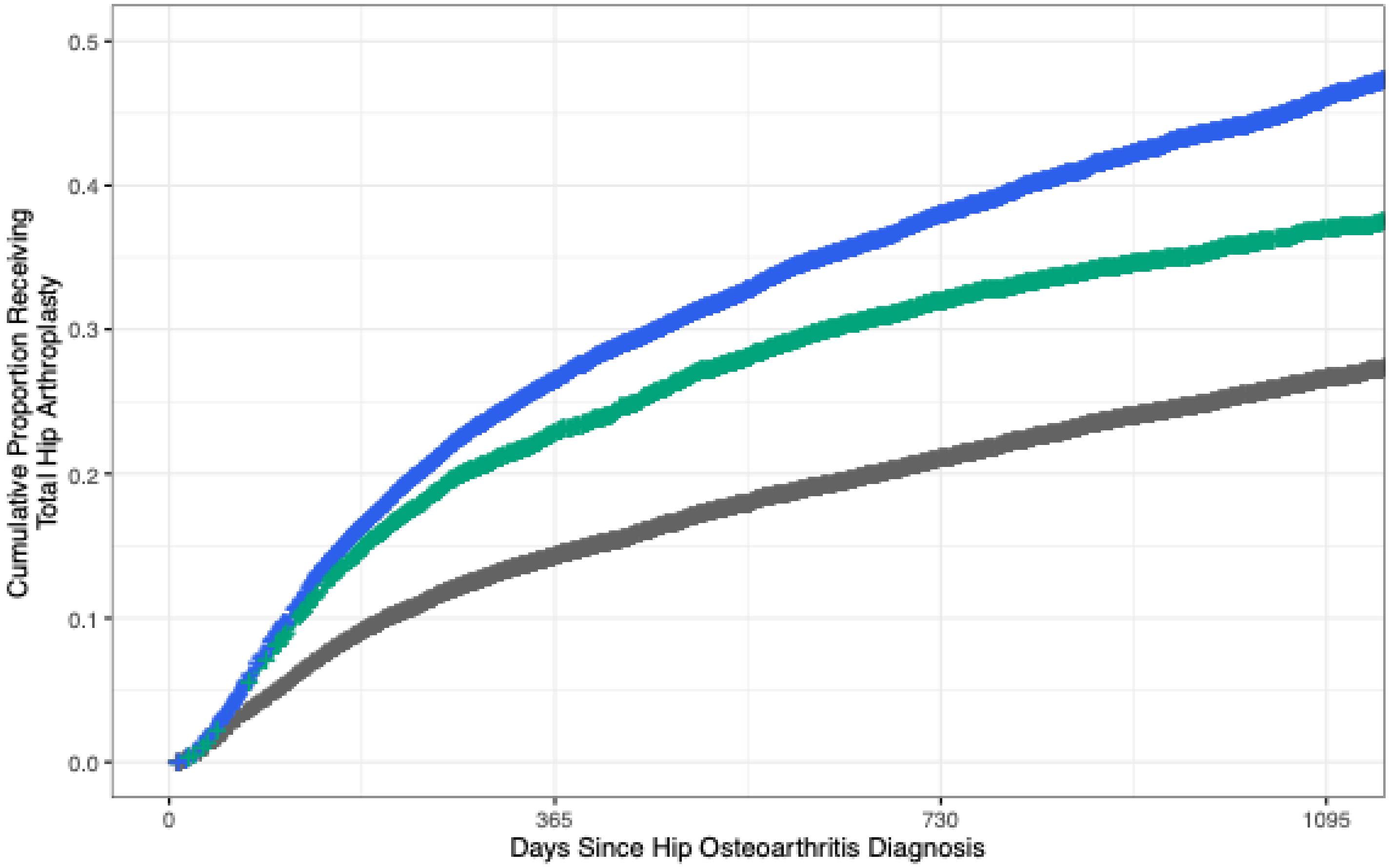

Number Uncensored

Age Group	0	365	730	1095
<55 Years	13325	10489	7984	5861
55–69 Years	17816	12683	9530	6917
70+ Years	6098	4455	3282	2423

Days Since Hip Osteoarthritis Diagnosis

Age Group: <55 Years, 55–69 Years, 70+ Years

© 2025 Hillery B et al. *JAMA Network Open*.

eFigure 1.e. Unadjusted survival curves and censoring frequency table by system of osteoarthritis diagnosis.

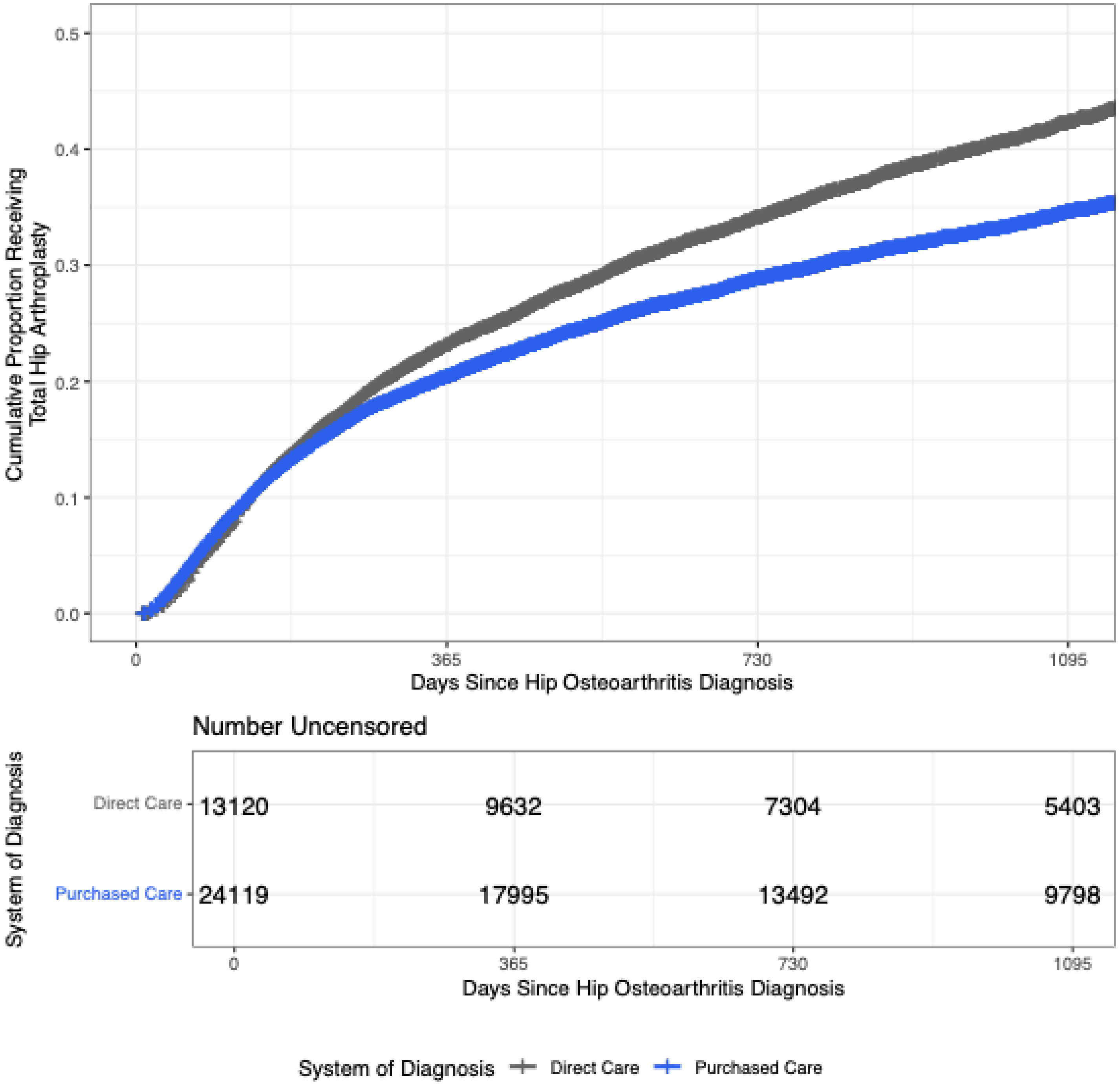

© 2025 Hillery B et al. *JAMA Network Open*.

eFigure 1.f. Unadjusted survival curves and censoring frequency table by geographic area.

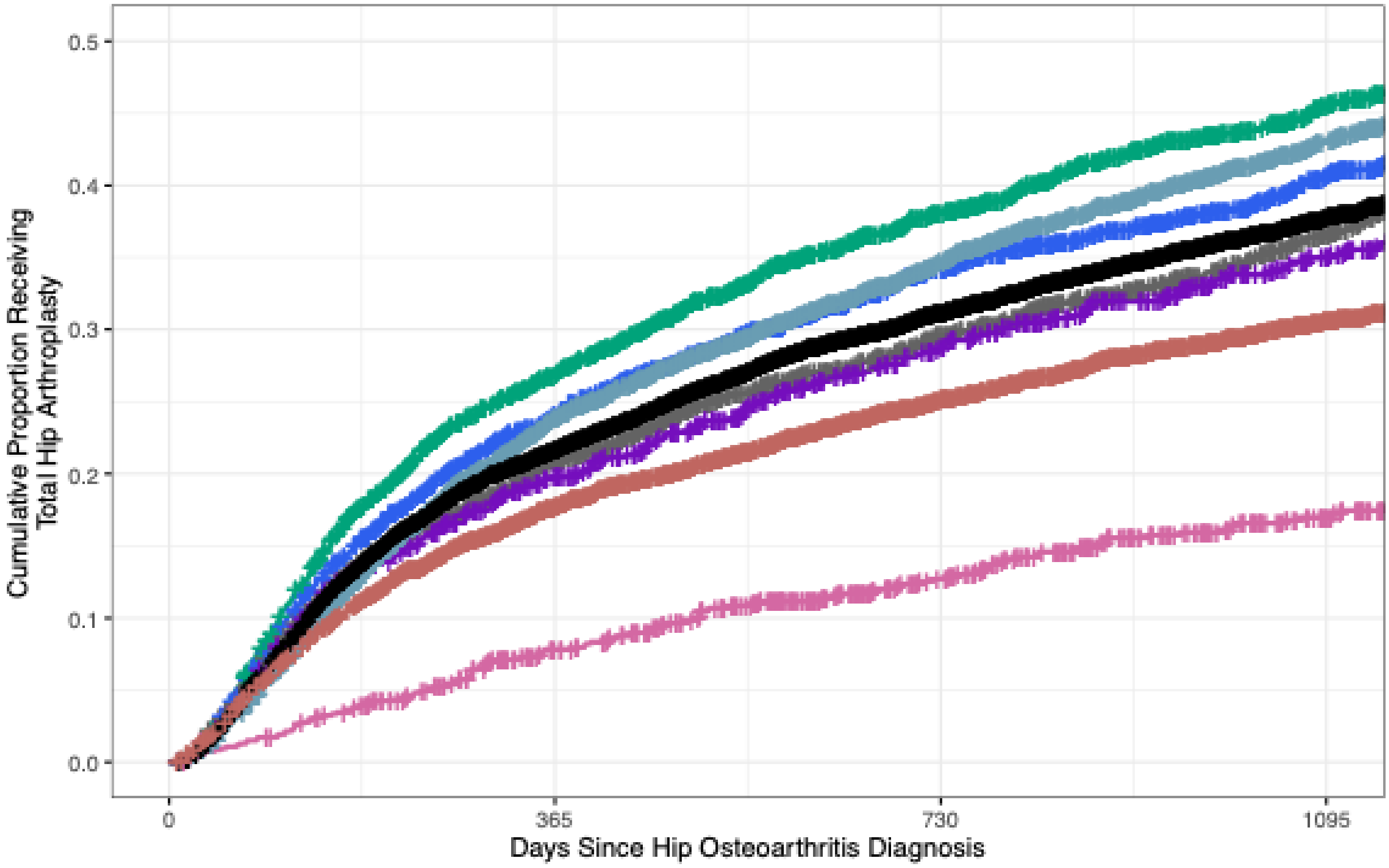

Number Uncensored

Geographic Area	0	365	730	1095
East South Central	2601	1922	1473	1081
Midwest	2873	2089	1571	1154
Mountain	3489	2462	1794	1321
Northeast	1196	897	683	492
US Territory, Another Country, Unknown	947	809	603	441
Pacific	4830	3518	2641	1922
South Atlantic	15059	11125	8275	6021
West South Central	6244	4805	3756	2769

Days Since Hip Osteoarthritis Diagnosis

Geographic Area: East South Central; Midwest; Mountain; Northeast; US Territory, Another Country, Unknown; Pacific; South Atlantic; West South Central

© 2025 Hillery B et al. *JAMA Network Open*.

eFigure 2. Covariate-adjusted probabilities (95%CI) of total hip arthroplasty receipt. Note: Adjusted probabilities were based on a priori reference categories of non-focal variables as follows: 60 years-old, male, active duty service member, with an age-adjusted Charlson Comorbidity Index (CCI) score of 1, no pre-index psychiatric or obesity diagnosis, with a pain-related diagnosis, index direct care system, index year 2020.6, 0 pre-index hip injections or imaging visits, 1 post-index imaging visit, 0 post-index opioid prescriptions, 2 post-index therapeutic visits, and South Atlantic geographic area.

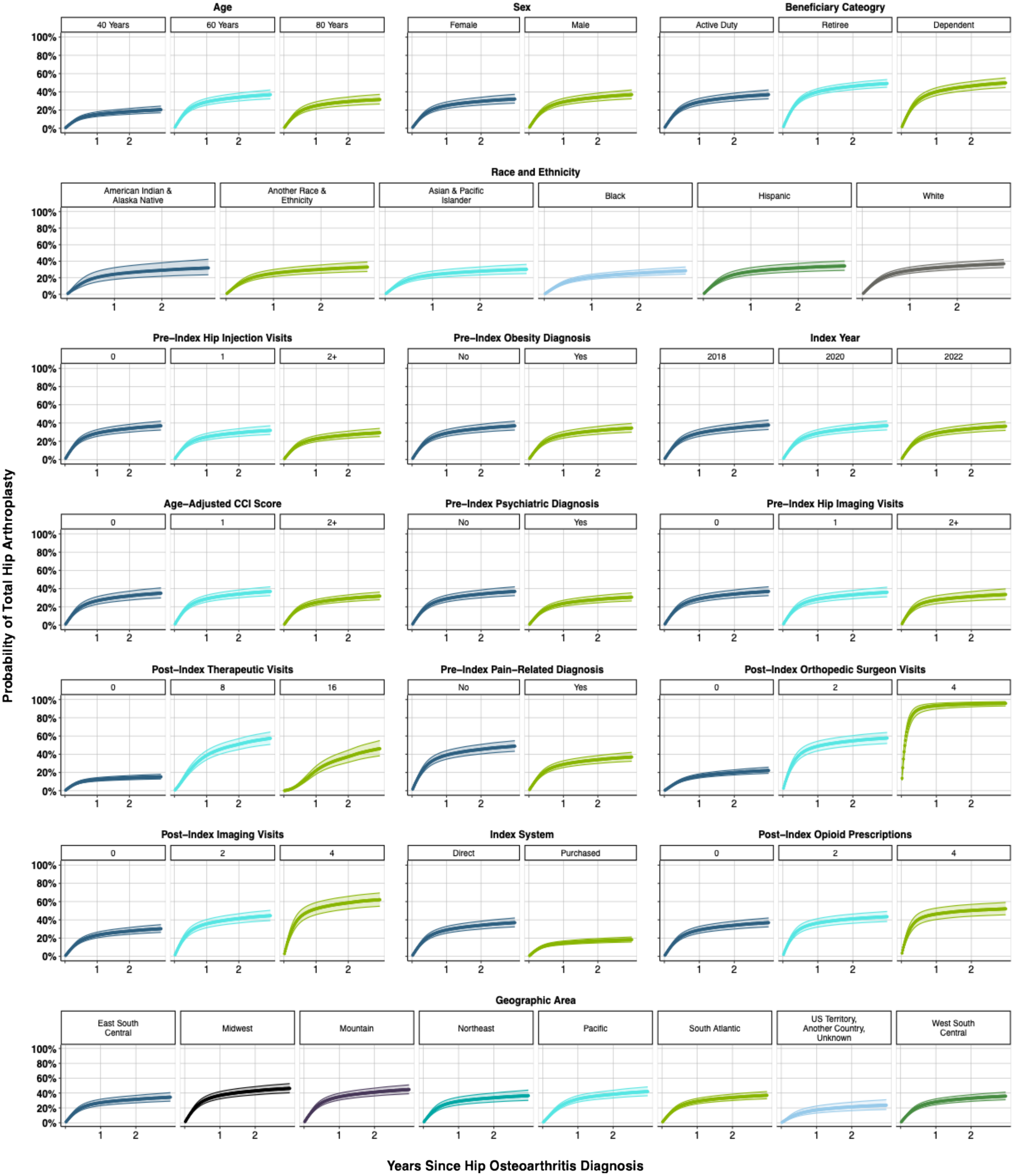

© 2025 Hillery B et al. *JAMA Network Open*.

eFigure 3. Cumulative hazard differences (95%CI) between comparator and reference groups for smooth and time-varying covariates in the primary model. Note: due to differences between the primary and sensitivity models, cumulative hazard differences across race and ethnicity and geographic area are depicted in eFigure 4 and eFigure 5, respectively.

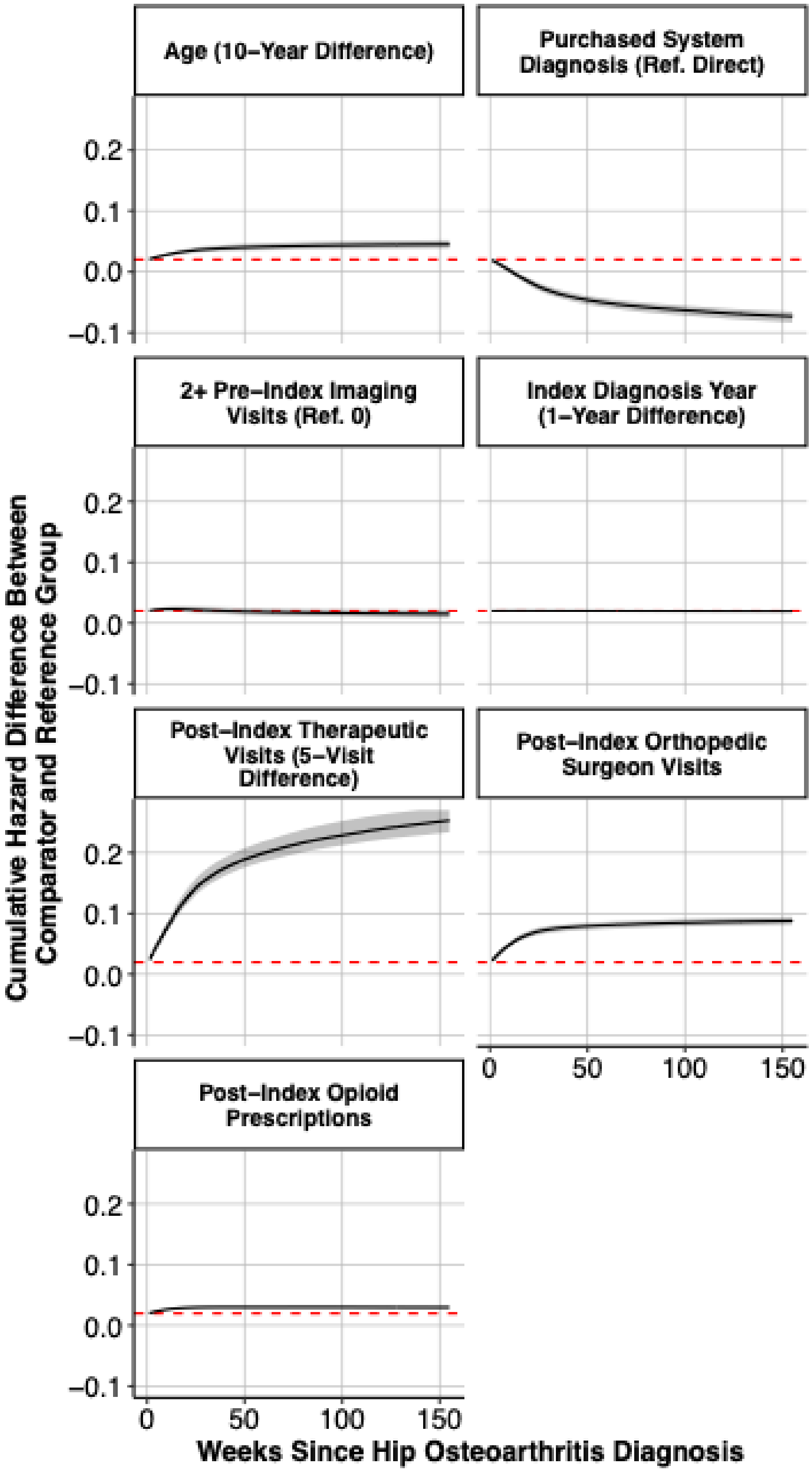

© 2025 Hillery B et al. *JAMA Network Open*.

eFigure 4. Cumulative hazard differences across race and ethnicity (columns) relative to white patients across the primary and three sensitivity models (rows). Note, values > 0.05 and < -0.05 were truncated to enable clarity in the overall pattern of effects. The y-axis scale varies across rows.

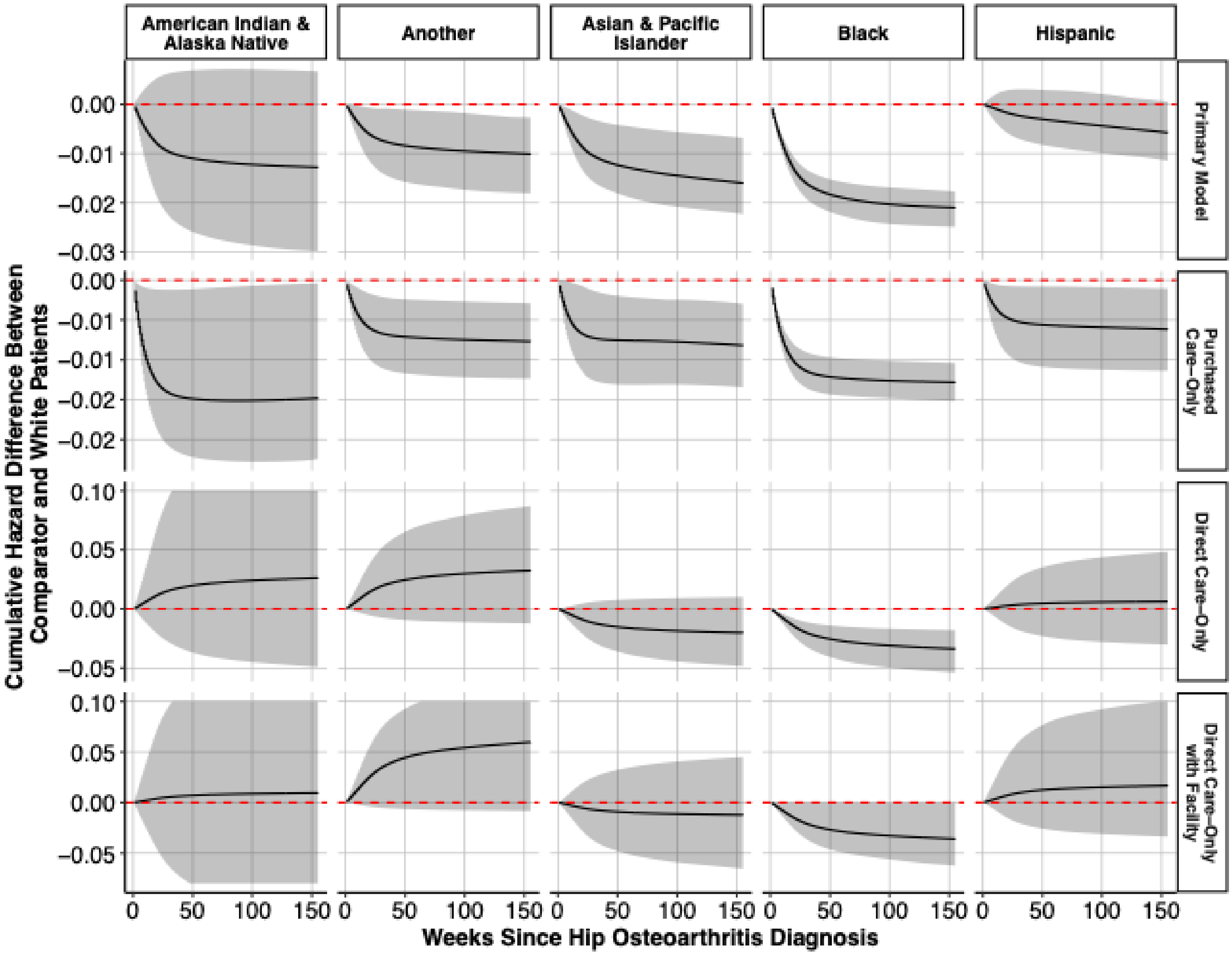

© 2025 Hillery B et al. *JAMA Network Open*.

eFigure 5. Cumulative hazard differences across geographic region (columns) relative to patients who received an index diagnosis in the South Atlantic region across the primary and three sensitivity models (rows). Note, values > 0.05 and < -0.05 were truncated to enable clarity in the overall pattern of effects.

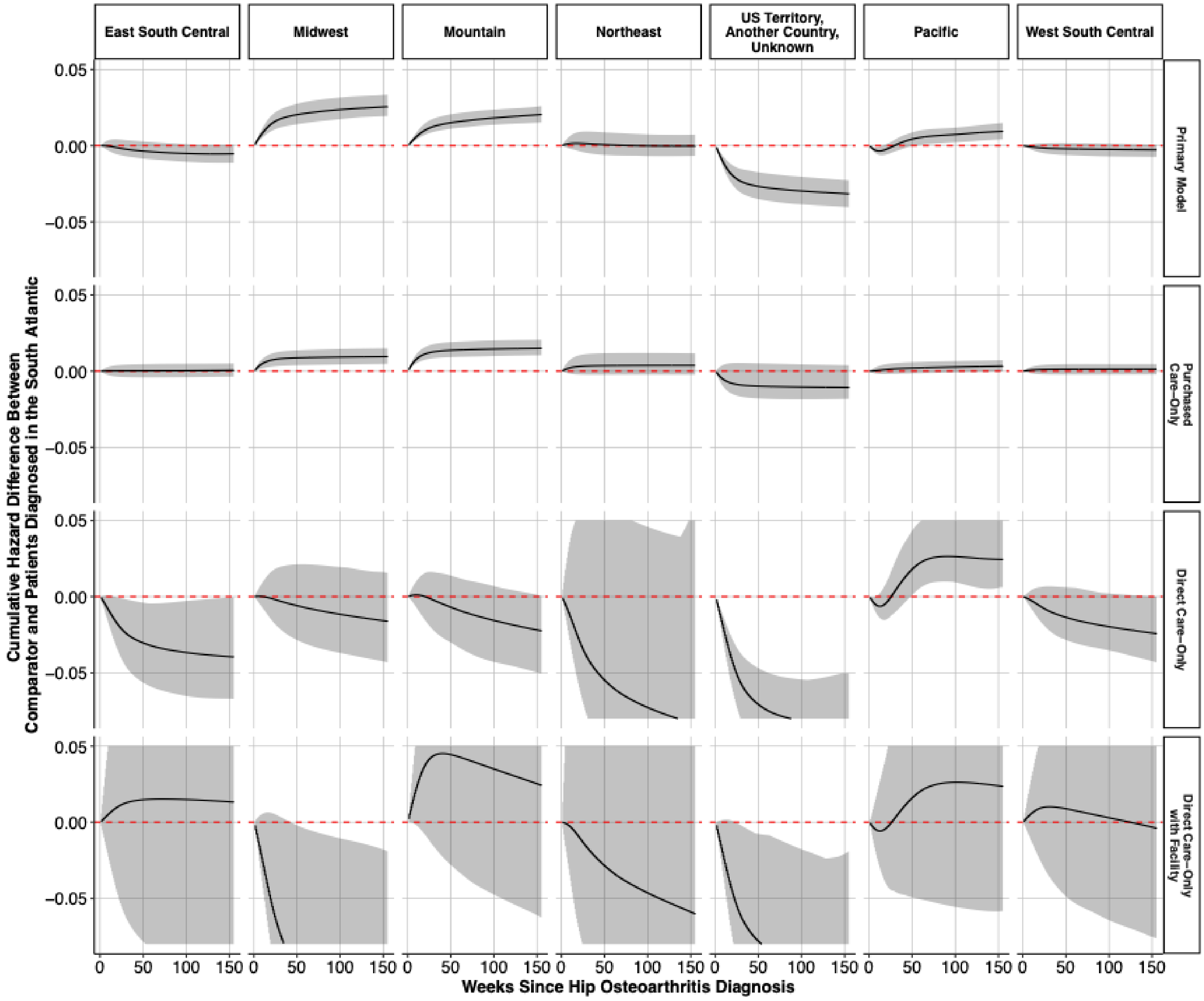

© 2025 Hillery B et al. *JAMA Network Open*.
