# Supplementary material for: Time to Total Hip Arthroplasty Among Patients in the US Military Health System
Source: JAMA Netw Open. 2025 Oct 28;8(10):e2539971. doi: 10.1001/jamanetworkopen.2025.39971 (PMC12569716; doi:10.1001/jamanetworkopen.2025.39971)
Supplement: Supplement 2. — Data Sharing Statement [file jamanetwopen-e2539971-s002.pdf]

Data Sharing Statement

Hillery. Time to Total Hip Arthroplasty Among Patients in the US Military Health System. *JAMA Netw Open*. Published October 28, 2025. doi:10.1001/jamanetworkopen.2025.39971

Data

Data available: No

Additional Information

Explanation for why data not available: The datasets generated and analyzed in this study are not publicly available due data sharing regulations and requirements of the US Department of Defense. Requests for data must be made directly to the US Defense Health Agency by investigators through data sharing agreement procedures, as indicated here: https://health.mil/Military-Health-Topics/Privacy-and-Civil-Liberties/Data-Sharing-Agreements.
